# Intra-Ramanome Correlation Analysis Unveils Metabolite Conversion Network from an Isogenic Population of Cells

**DOI:** 10.1128/mBio.01470-21

**Published:** 2021-08-31

**Authors:** Yuehui He, Shi Huang, Peng Zhang, Yuetong Ji, Jian Xu

**Affiliations:** a Single-Cell Center, CAS Key Laboratory of Biofuels, Shandong Key Laboratory of Energy Genetics and Shandong Energy Institute, Qingdao Institute of BioEnergy and Bioprocess Technology, Chinese Academy of Sciences, Qingdao, Shandong, China; b Laboratory for Marine Biology and Biotechnology, Qingdao National Laboratory for Marine Science and Technology, Qingdao, Shandong, China; c University of Chinese Academy of Sciences, Beijing, China; d Department of Pediatrics and Center for Microbiome Innovation at Jacobs School of Engineering, University of California San Diego, La Jolla, California, USA; Korea Advanced Institute of Science and Technology

**Keywords:** ramanome, intra-ramanome correlation analysis (IRCA), intra-ramanome correlation network (IRCN), single-cell Raman spectroscopy, phenotypic heterogeneity

## Abstract

To reveal the dynamic features of cellular systems, such as the correlation among phenotypes, a time or condition series set of samples is typically required. Here, we propose intra-ramanome correlation analysis (IRCA) to achieve this goal from just one snapshot of an isogenic population, via pairwise correlation among the cells of the thousands of Raman peaks in single-cell Raman spectra (SCRS), i.e., by taking advantage of the intrinsic metabolic heterogeneity among individual cells. For example, IRCA of Chlamydomonas reinhardtii under nitrogen depletion revealed metabolite conversions at each time point plus their temporal dynamics, such as protein-to-starch conversion followed by starch-to-triacylglycerol (TAG) conversion, and conversion of membrane lipids to TAG. Such among-cell correlations in SCRS vanished when the starch-biosynthesis pathway was knocked out yet were fully restored by genetic complementation. Extension of IRCA to 64 microalgal, fungal, and bacterial ramanomes suggests the IRCA-derived metabolite conversion network as an intrinsic metabolic signature of isogenic cellular population that is reliable, species-resolved, and state-sensitive. The high-throughput, low cost, excellent scalability, and general extendibility of IRCA suggest its broad applications.

## INTRODUCTION

Each isogenic population of cells is characterized by many phenotypes that change with time and condition. Such phenotypes can be related to cellular metabolism, such as the amount of a particular metabolite produced under a given time and condition. Links among such metabolism-related phenotypes are a fundamental property that underlies proper functioning of cellular systems (1–3). For example, correlation in abundance among metabolites can unravel, via metabolome-wide association studies (MWAS) of metabolomic data sets from high-resolution mass spectrometry (MS), the links among target metabolites that characterize a certain disease (4–6). Therefore, strategies and methods for rapidly and comprehensively detecting and profiling such links are of interest.

To detect such links, variations in metabolism-related phenotypes are required. As a result, typically, a time series or condition series set of samples is characterized for metabolite contents first and then correlated across the samples, with the resolution of detection generally dependent on sample size (7–9). For example, in order to understand the mechanism of oil production in microalgae, the lipidomes of an industrial microalga, Nannochloropsis oceanica, were measured in triplicate by electrospray ionization MS over eight time points (3, 4, 6, 12, 24, 48, 72, and 96 h) under nitrogen-replete (N+) or nitrogen-depleted (N–) conditions; then the profiles were correlated across the 48 samples, which revealed several temporal patterns among the triacylglycerol (TAG) species that suggest distinct regulation mechanisms; e.g., TAGs with lower degrees of desaturation were induced at the early stages of N–, while those containing polyunsaturated fatty acids (PUFAs) increased considerably only at later stages (10).

However, within a single sample of an isogenic population of cells, at any given time or condition, variations in metabolism-related phenotypes among individual cells are inherent and universal, due to the stochasticity of gene expression in cells (11, 12). Therefore, an intriguing question is, can such phenotypic variations among individual cells, instead of those among multiple samples, be exploited to predict the links among metabolism-related phenotypes? Specifically, there are three hypotheses: (i) every cell can be quantitatively profiled for its various metabolism-related phenotypes as an independent biological replicate; (ii) intercellular variations of the phenotypes can be measured rapidly and simultaneously for many cells; (iii) intercellular correlation of the phenotypes can unveil important functional features of the system.

To test these hypotheses, we employed single-cell Raman microspectrometry, which captures the *in vivo* chemical profiles of a cell in a rapid, label-free, and nondestructive manner (13–15). In a single-cell Raman spectrum (SCRS) (13–15), each of (or a combination of) its thousands of Raman peaks potentially represents a specific metabolism-related phenotype, such as the presence and the concentration of a metabolite synthesized by the cell (16). Therefore, just like a portrait can reveal multiple facial features from a human individual, an SCRS can unveil cellular phenotypes (i.e., functions) in a “landscape-like” manner, i.e., simultaneously revealing multiple metabolism-related phenotypes of the cell at that particular state (16). For example, in a single microalgal cell (e.g., Chlamydomonas reinhardtii and *N. oceanica*), starch content can be quantified by the specific Raman peaks of 478 cm^−1^ (C-C-C deformation) and 940 cm^−1^ (C-O stretching; C-O-C and C-O-H deformation; α-helix C-C backbone), while the content of triacylglycerol (TAG) can be modeled by 1,441 cm^−1^ (alkyl C-H_2_ bend) and 2,851 cm^−1^ (C-H_2_; C-H_3_ asymmetric and symmetric stretches) (17, 18). In addition, the degree of lipid unsaturation in a cell can be measured by I_1,658_/I_1,441_, i.e., the ratio of 1,658 cm^−1^ (allyl C=C stretches which are proportional to the amount of unsaturated C=C bonds) and 1,441 cm^−1^ (alkyl C-H_2_ bends which are proportional to the amount of saturated C-C bonds) (18, 19). On the other hand, based on the full spectra of SCRS, the contents of many compounds in a cell can be derived, such as protein, starch, and TAG in C. reinhardtii (18) and astaxanthin, and β-carotene and chlorophyll in Haematococcus pluviali (20), via chemometric multivariate methods including partial least square regression (PLSR) and multivariate curve resolution (MCR).

**FIG 1:**
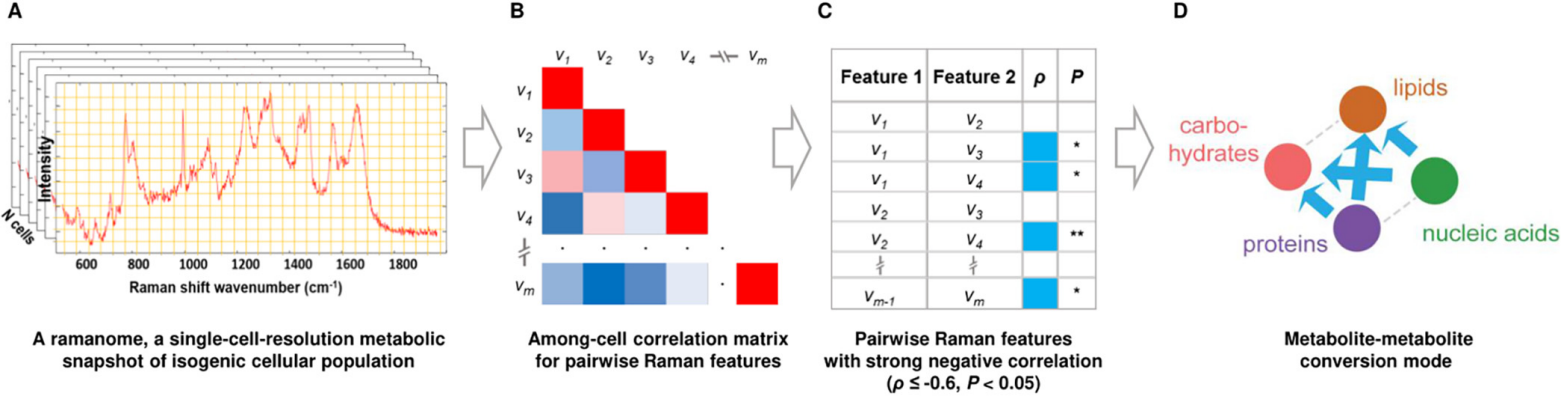
The principle and workflow of intra-ramanome correlation analysis (IRCA).

We previously proposed the “ramanome” concept (16, 21). A ramanome is the collection of SCRS (one from each cell) randomly sampled from a given instance of an isogenic cellular population and thus represents a single-cell-resolution metabolic snapshot of the population (16, 21). Here, by treating every SCRS in a ramanome as one biological replicate and each of its peaks or combination of peaks as a metabolism-related phenotype, we established a formal framework called intra-ramanome correlation analysis (IRCA; Fig. 1). From just one single ramanome, by pairwise correlating all the Raman peaks of SCRS among the individual cells, IRCA unveils a network of potential metabolite conversions. Based on a series of ramanome-profiling experiments on C. reinhardtii mutants and additional microalgae, fungi, and bacteria, we showed that IRCA is a rapid, low-cost, high-throughput, landscape-like, and universally applicable method for unraveling metabolic features of cellular systems.

## RESULTS

### IRCA predicts the interconversion among starch, protein, and TAG from one single instance of an isogenic microalgal population.

**FIG 2:**
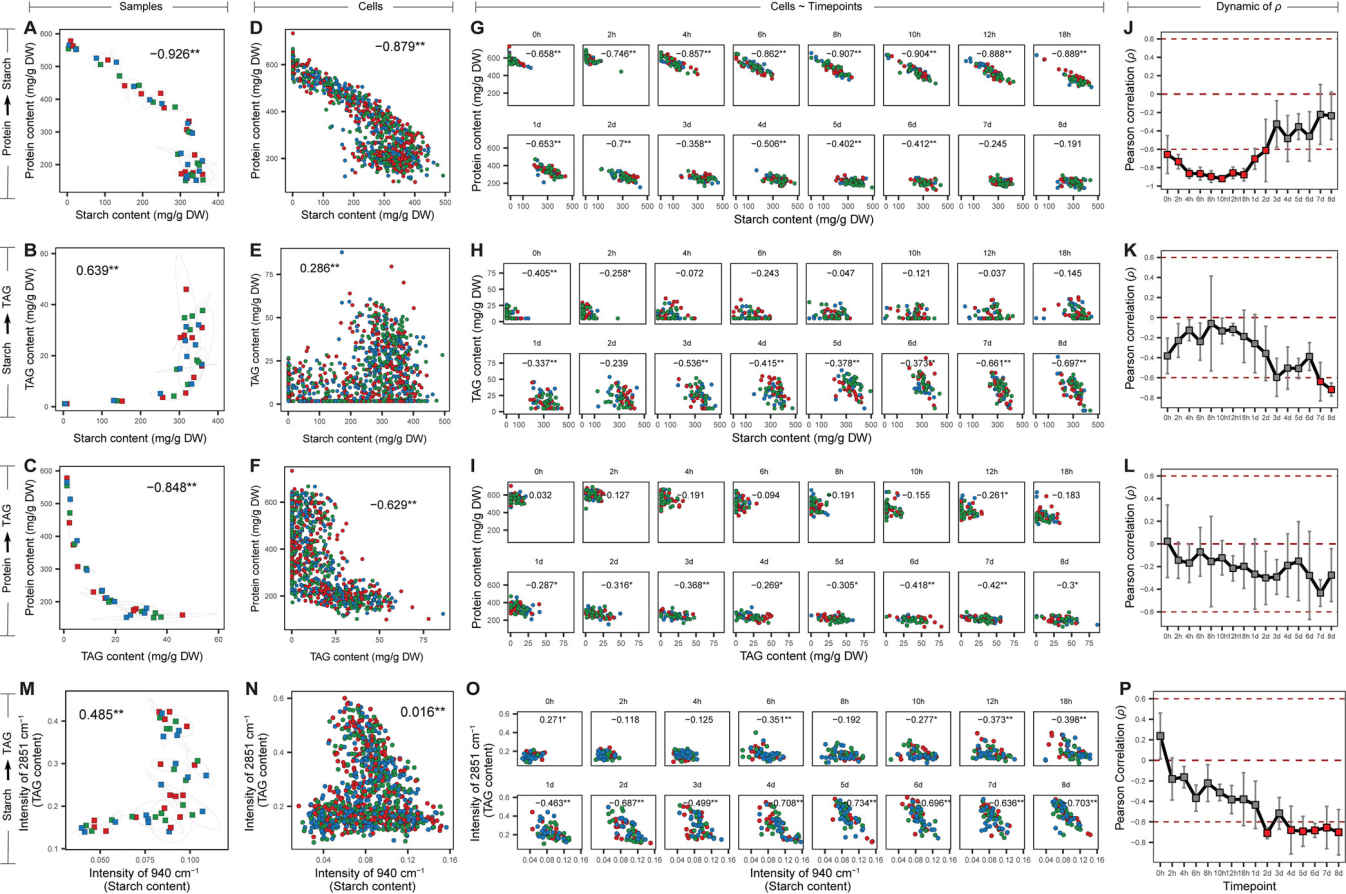
Correlation of starch and TAG contents of the wild-type C. reinhardtii CC124 at the population level and the single-cell level. (A to F) Correlation of starch and total protein contents at the population level (A) and the single-cell level (D) was shown; so was that of starch and TAG contents at the population level (B) and the single-cell level (E) and that of TAG and total protein contents at the population level (C) and the single-cell level (F). Each dot in panels A, B, and C represent one sample. Each dot in panels D, E, and F represent one individual cell. (G to L) Correlation of starch and TAG contents modeled by multiple pairs of singular Raman peaks among individual cells at each time point. Phenotypic correlation between starch (*x*) and protein (*y*) contents (G and J), between starch (*x*) and TAG (*y*) contents (H and K), and between TAG (*x*) and protein (*y*) contents (I and L) are also shown. (M to P) Phenotypic correlation between starch (*x*) and TAG (*y*) contents modeled by pair of Raman peaks of 940 cm^−1^ and 2,851 cm^−1^. Correlation of the starch and TAG contents at the population level (M) and single-cell level (N) was compared. Phenotypic correlation between starch (*x*) and TAG (*y*) contents (O and P) at each time point were shown. In the curves of temporal dynamics, *ρ* is the Pearson correlation coefficients of two phenotypes among single cells (**, *P < *0.01; *, *P < *0.05), with value indicating the mean of triplicates and the error bar indicating the standard deviation. Absence of correlation (*ρ* = 0) or presence of strong correlation (*ρ* ≥ 0.6 or *ρ* ≤ –0.6) is highlighted by red horizontal lines.

To test the IRCA concept (Fig. 1), we employed the unicellular microalga of C. reinhardtii CC124 as the first model. Triplicate isogenic cultures of CC124 grown under nitrogen depletion (N–) were sampled from 16 time points from 0 h to 8 days (i.e., 48 ramanomes in total; see Fig. S1; Materials and Methods). For cellular protein, starch, and TAG contents, each sample was analyzed via two strategies: (i) at the population level, via various conventional techniques which all require metabolite extraction from cell lysates (bicinchoninic acid [BCA] protein assay kit, amyloglucosidase/α-amylase method and thin-layer chromatography plus gas chromatography/mass spectrometry [TLC-GC-MS]; Fig. 2A to C; also see Text S1) and (ii) at the single-cell level, via full-SCRS-based quantitative partial least square regression (PLSR) models for metabolite contents, which are noninvasive and label-free (18) (∼20 randomly selected cells per sample and thus 60 per time point; Fig. 2D to F). The PLSR model for starch content, for example, was established using the population-level measurements (i.e., via the amyloglucosidase/α-amylase method; see Text S1) and the averaged SCRS of 20 cells in the corresponding biological replicate (i.e., one ramanome). The correlation coefficients (*R*^2^) of protein, starch, and TAG contents between the two strategies above are 0.9924, 0.9892, and 0.9686, respectively, confirming high accuracy of simultaneous quantification of protein, starch, and TAG for a C. reinhardtii cell via its full SCRS (18). Notably, for each phenotype, at any instance of the population, the degree of intercellular heterogeneity is high, and can vary greatly along the 8 days (see Fig. S1G to I). Specifically, for protein, both minimal and maximal single-cell contents in the population were decreasing with time (see Fig. S1G), while for starch, the trends are exactly the opposite (see Fig. S1H). As for TAG, the maximal content was increasing, while the minimal content remained at the baseline (i.e., a subpopulation of non-TAG-producing cells was always present), with the degree of within-population heterogeneity continuing to grow (i.e., the “delta” in Fig. S1I).

10.1128/mBio.01470-21.1 TEXT S1 Supplemental materials and methods for (i) the quantification of starch, protein, and TAG contents (at the population level) for the Chlamydomonas reinhardtii wild-type strain CC124, (ii) quantifying the influence of sampling depth on intra-ramanome correlation analysis. Supplemental results showing the effect of sampling depth on intra-ramanome correlation analysis. Download Text S1, DOC file, 0.3 MB. Copyright © 2021 He et al. 2021 He et al. https://creativecommons.org/licenses/by/4.0/ This content is distributed under the terms of the Creative Commons Attribution 4.0 International license.

10.1128/mBio.01470-21.2 FIG S1 Dynamics of the starch, protein, and TAG contents at the population level and at the single-cell level. (A to F) The parameters were measured for the populations via conventional approaches (A to C) or for single cells via prediction by ramanome (D to F). The max, min, and delta of contents for each timepoint are also shown (G to I). Download FIG S1, TIF file, 1.5 MB. Copyright © 2021 He et al. 2021 He et al. https://creativecommons.org/licenses/by/4.0/ This content is distributed under the terms of the Creative Commons Attribution 4.0 International license.

Notably, at the population level, significant correlation (defined by the Pearson correlation coefficient, or *ρ*) was observed in each of three phenotype pairs among the 48 populations—protein-starch (*ρ* = –0.926; Fig. 2A), starch-TAG (*ρ* = 0.639; Fig. 2B), and protein-TAG (*ρ* = –0.848; Fig. 2C). This indicates protein-starch conversion, protein-TAG conversion, and accordant change of starch and TAG contents (22, 23). Intriguingly, at the single-cell level, correlation for the 960 cells collectively reached a consistent conclusion, albeit with lower correlation coefficients (*ρ* = –0.879, 0.286, and –0.229, respectively; Fig. 2D to F). Thus, the interphenotype correlation among pooled cells from all populations can recapitulate that among the populations.

We then probed whether such interphenotype correlation can be detected via cells from just one instance of a population, i.e., at each of the 16 time points (Fig. 2G to I). Intriguingly, for protein-starch, negative correlation (NC) is phase-specific and shows a temporal trend of gradual weakening, quite strong at each of the time points only before 2 days (*ρ* ≤ –0.6, *P < *0.01; Fig. 2G) yet absent at 7 days or 8 days (Fig. 2J). Thus, it is possible that the protein-starch conversion took place only at the early phase of N–. In fact, this prediction is supported by the routing of hydrolytic release of carbon skeletons from protein to the synthesis of starch granules during the first 2 days in C. reinhardtii under N–, as previously observed (22, 23).

For starch-TAG, the situation is the opposite, as the trend of NC among cells within a population intensified along time (Fig. 2H and K). Thus, in contrast to the positive correlation (PC) between starch and TAG at the population level (*ρ* = 0.639, *P < *0.01; Fig. 2B), the within-population NC between starch and TAG was prominently present at the late phase (i.e., starting from 3 days; Fig. 2K), which predicts conversion between starch and TAG inside the cell. This single-cell-based prediction, which the population-level analysis missed, is actually supported by two observations: (i) competition between starch and lipid synthesis for their shared biosynthetic precursors intensifies temporally (24–27), and (ii) early build-up of starch may serve as the carbon source for lipid synthesis at a later subsequent phase (28–32).

For protein-TAG, unlike their very strong among-population NC (*ρ* = –0.848, *P < *0.01; Fig. 2C), no strong correlation was found for each population, although the trend of NC gradually intensified (Fig. 2I and L). In particular, at 2 days and beyond, the protein contents of individual cells were all already depleted to a very low level, despite their relatively wide range of TAG contents, which indicates the decoupling of protein-synthetic and TAG-synthetic pathways. This distinction in the temporal within-population NC pattern between protein-starch (Fig. 2J) and protein-TAG (Fig. 2L) suggests that (i) prior to 2 days, the majority of released carbon skeletons from proteins were routed to starch rather than to lipids and (ii) when starch biosynthesis was saturated at 2 days, substantial accumulation of TAG occurred which is converted partially from proteins (23). Therefore, by correlating SCRS-derived phenotypes among cells, IRCA can reveal intermetabolite conversions from just one instance of an isogenic population.

Notably, besides the full SCRS (18), individual Raman peaks can also quantitatively model single-cell starch, protein, and TAG contents. For example, 940 cm^−1^ (C-O stretching, C-O-C, C-O-H deformation, and α-helix C-C backbone) and 2,851 cm^−1^ (C-H_2_, C-H_3_ asymmetric and symmetric stretches) can model starch and TAG contents, respectively (the correlation coefficient *R*^2^ between bulk-biomass-based conventional method and SCRS-based method being 0.884 and 0.954, respectively; see Fig. S2A and B). Moreover, correlations via just these two peaks among populations (Fig. 2M) or among cells (Fig. 2N) are consistent with those based on the full SCRS (Fig. 2B and E). When using only these two peaks to model single-cell starch and TAG, strong within-population starch-TAG NC was absent at the early stage of N– (0 h to 1 day, 3 days) yet emerged at the later stage of N– (2 days, 4 days to 8 days; Fig. 2O and P). These results from C. reinhardtii CC124, which are consistent with the full-SCRS-derived findings described above, raise the possibility of reconstructing a network of potential correlations among metabolites by (i) treating the intensity of each peak as the content of a potential metabolite (or class of metabolites), and (ii) pairwise correlating all the ∼1,600 peaks in an SCRS among the cells in a ramanome.

10.1128/mBio.01470-21.3 FIG S2 Validation of the switch between the starch and TAG contents based on wild-type and mutant strains of the laboratory model microalga of C. reinhardtii. (A and B) Phenotypic correlations between the starch and TAG contents measured by conventional methods and via intensity of 940 cm^−1^ (A) and 2,851 cm^−1^ (B) in SCRS were compared. (C to K) Phenotypic correlations between starch (*x*) and TAG (*y*) contents modeled by pairs of 940 cm^−1^ and 2,851 cm^−1^, respectively, for CC124 (C), CC4325 (D), CC406 (E), CC4324 (F), CC4326 (G), CC4333 (H), CC4334 (I), CC4565 (J), and CC4566 (K) of C. reinhardtii strains are shown. Detailed genotypes of the strains are listed in **Table S1**. The starch and TAG contents and Pearson correlation coefficients among the phenotypes among single cells within one ramanome are shown with a heatmap (**, *P < *0.01; *, *P < *0.05). Download FIG S2, TIF file, 2.0 MB. Copyright © 2021 He et al. 2021 He et al. https://creativecommons.org/licenses/by/4.0/ This content is distributed under the terms of the Creative Commons Attribution 4.0 International license.

### Genetically validating IRCA by knockout and then complementation of starch synthetic genes.

**FIG 3:**
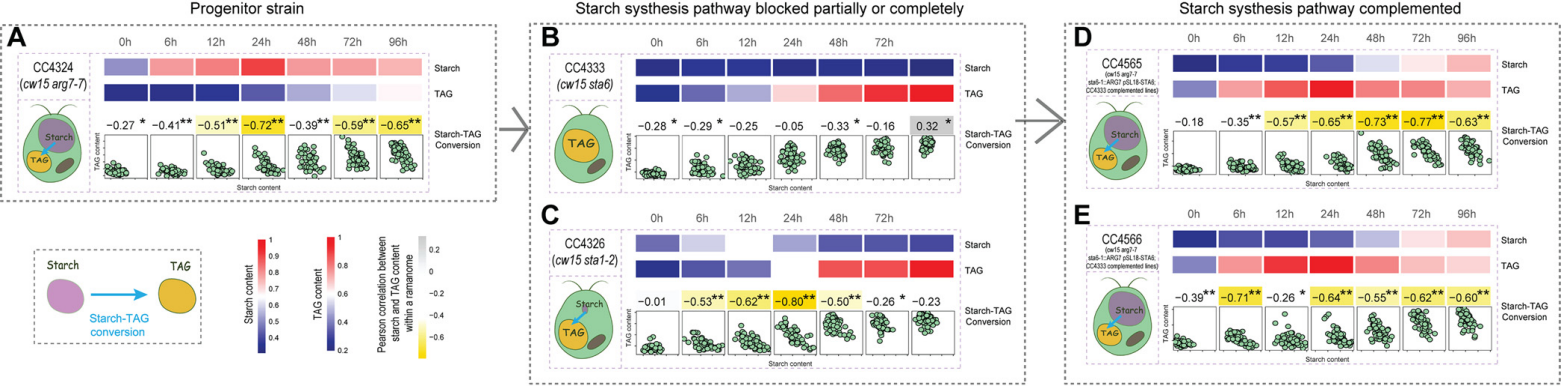
Validation of the IRCA method via a genetic approach using a series of targeted mutants of C. reinhardtii. Correlation between starch (*x*) and TAG (*y*) contents (modeled by 940 cm^−1^ and 2,851 cm^−1^, respectively) among cells within a ramanome for the mutant strains (Table S1)—CC4324 (A), CC4333 (B), CC4326 (C), CC4565 (D), and CC4566 (E). The starch and TAG contents and their Pearson correlation coefficients are shown via heatmap (**, *P < *0.01; *, *P < *0.05). Each dot in the scatterplots represents one cell.

To validate such IRCA-based prediction of intermetabolite conversions, we employed a genetic approach (see Fig. S2C to K; Fig. 3; also see Table S1). CC4325, a mutant directly derived from CC124 by X-ray mutagenesis, is deficient of starch due to the knockout of *sta1-1* (which contributes to starch synthesis by catalyzing synthesis of the activated glycosyl donor, ADP-glucose, from Glc-1-P and ATP [33]), and thus, the starch-TAG conversion (i.e., NC between starch and TAG peaks in IRCA) should attenuate in this mutant. As expected, IRCA of the time series CC4325 ramanomes revealed that the strong starch-TAG NC among single cells was no longer present (0 to 96 h under N–; see Fig. S2D), in contrast to CC124 (Fig. 2O and P). To validate the specificity of IRCA in revealing the starch-TAG conversion, we further collected the time series ramanomes for CC406, a wall-less strain derived from CC124 (34). For CC406, IRCA revealed strong NC (*ρ* ≤ –0.6) starting at 6 h under N– and remained so afterwards, suggesting starch-to-TAG conversion (see Fig. S2E). Therefore, the cell wall does not interfere with the detection of starch-TAG conversion via IRCA.

10.1128/mBio.01470-21.10 Table S1 Microalgal and microbial strains, culture conditions, and time points sampled for IRCA in this study and assignments of the Raman peaks used for deriving the metabolite profile in this study. Download Table S1, DOC file, 0.1 MB. Copyright © 2021 He et al. 2021 He et al. https://creativecommons.org/licenses/by/4.0/ This content is distributed under the terms of the Creative Commons Attribution 4.0 International license.

Similarly, for CC4324 (i.e., *cw15 arg7-7*), a cell wall-deficient, arginine-requiring and starch-producing strain derived from CC406, significant NC took place throughout the 96 h (especially at 24 h, 72 h, and 96 h; Fig. 3A). In contrast, for CC4333 (i.e., *sta6-1*; Fig. 3B) or CC4334 (i.e., *sta7-1*; see Fig. S3I), which both are direct CC4324 derivatives yet produce no starch due to disruption of *sta6* (ADP-glucose pyrophosphorylase) (35) and *sta7* (isoamylase) (36), respectively (34), no strong starch-TAG correlation (*ρ* > –0.6) was reported by IRCA over the full course (0 to 96 h under N–).

10.1128/mBio.01470-21.4 FIG S3 Validation of IRCA based on the presence and absence of starch-TAG conversion in a series of targeted mutants of C. reinhardtii. (A, C, and E) Phenotypic correlation between starch (*x*) and TAG (*y*) contents modeled by a pair of Raman peak of 940 cm^−1^ and 2,851 cm^−1^ for CC4326 (A), CC4333 (C), and CC4326 (E). (B, D, and F) In the scatterplots, each dot represents one cell. In the curves of temporal dynamics, *ρ* is the Pearson correlation coefficient between two phenotypes among single cells (**, *P < *0.01; *, *P < *0.05), with the value indicating the mean of triplicates and the error bar indicating the standard deviation for CC4326 (B), CC4333 (D), and CC4334 (F). The absence of correlation (*ρ* = 0) or presence of strong correlation (*ρ* ≥ 0.6 or *ρ* ≤ –0.6) is highlighted by red horizontal lines. Detailed genotypes of the strains are listed in **Table S1**. Download FIG S3, TIF file, 1.1 MB. Copyright © 2021 He et al. 2021 He et al. https://creativecommons.org/licenses/by/4.0/ This content is distributed under the terms of the Creative Commons Attribution 4.0 International license.

Interestingly, for CC4326 (i.e., *sta1-2*), another direct CC4324 derivative whose starch synthesis pathway is only partially disrupted (34), the temporal correlation pattern in IRCA is distinct from CC4333 and CC4334, which are fully starchless (under N–; Fig. 3C); instead of the latter’s full ablation of starch-TAG NC throughout 96 h, CC4326 exhibited strong starch-TAG NC, yet only at the middle phase of from 12 h to 48 h, consistent with transient accumulation and conversion of a low level of starch to TAG, and then ceased the conversion upon starch depletion. Notably, under N+, which serves as a control condition (no TAG is accumulated in C. reinhardtii under abundant medium N [34]), CC4326 did not exhibit the starch-TAG NC until at the very late phase (e.g., 96 h, when the medium N was depleted by algal growth); in contrast, for CC4333 and CC4334, no starch-TAG NC was apparent throughout the 96 h under N+, consistent with their genotypes (i.e., complete disruption of the starch synthetic pathway; see Fig. S3 [34]).

Importantly, for both CC4565 and CC4566, both genetically complemented strains with fully restored starch synthetic capability (i.e., the *sta6* gene was reintroduced into CC4333 by overexpression via pSL18-STA6 [34]), the temporal patterns of starch-TAG NC under N– are identical to those of CC4324, which is the direct progenitor of CC4333 and carries an intact starch synthetic pathway; they all exhibited significant NC throughout the 96 h (under N–; Fig. 3D and E). Altogether, the starch-TAG NC in IRCA that indicates starch being converted to TAG is directly correlated with starch-synthetic genotypes. Therefore, IRCA can detect and model the interconversion between metabolites from a single instance of isogenic population.

### IRCNs reveal novel product-product and substrate-product links from an isogenic population.

**FIG 4:**
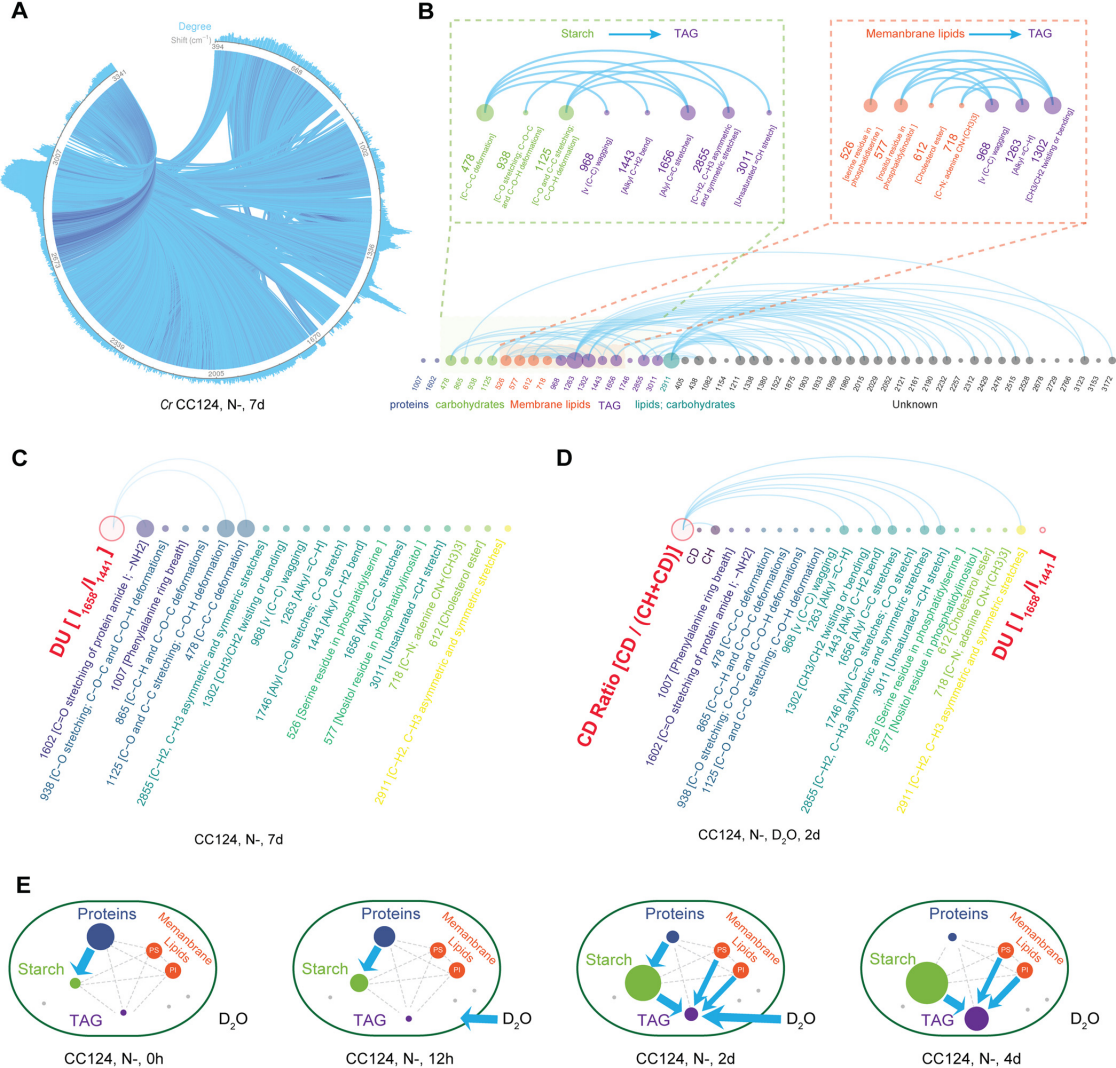
Global and local IRCNs of the wild-type C. reinhardtii. (A) The global IRCN of 1,581 Raman peaks of CC124 under N– at 7 days (*ρ* ≤ –0.6, *P < *0.05). (B) The local IRCN with 51 Raman peaks of CC124 under N– at 7 days. (C) The local IRCN (i.e., 17 characteristic Raman peaks) that includes the DU of CC124 under N– at 7 days (*ρ* ≤ –0.6, *P < *0.05). (D) The local IRCN that further includes the CD ratio of CC124 under 25% D_2_O and N– at 2 days. Edges represent strong negative correlations (*ρ* ≤ –0.6, *P < *0.05). N–, nitrogen-depleted condition. (E) Key substrate-product or product-product links discovered by IRCNs. Contents of starch and TAG were quantified by 940 cm^−1^ and 2,851 cm^−1^, respectively. Protein content was modeled via the full SRCS. The degree of DU was quantified by the ratio between 1,658 cm^−1^ (unsaturated C=C bonds) and 1,441 cm^−1^ (saturated C-C bonds). The D_2_O incorporation rate was quantified by the CD ratio, i.e., the ratio between the C-D bond area (2,040 to 2,300 cm^−1^) and the area of C-D plus C-H bonds (2,040 to 2,300 cm^−1^ and 2,800 to 3,050 cm^−1^). Blue lines and blue arrows represent strong conversions.

In a ramanome, the 1,581 Raman peaks in each of the many SCRS represent over one million pairwise correlations among individual cells. Such richness of information indicates an enormous number of possible between-phenotype links (and, in particular, between-metabolite conversions). For example, for the CC124 N– 7-day ramanome that consists of 60 cells (which exceeds the minimal sampling depth for IRCA; see Text S1; also see Fig. S4), all pairwise among-cell correlations of peaks unveiled, in total, 60,994 strong NC “links” (*ρ* ≤ –0.6, *P < *0.05) that formulate a 1,581-node network (Fig. 4A; Materials and Methods). In such an intra-ramanome correlation network (IRCN), a node represents a Raman peak in SCRS and thus a potential phenotype (e.g., a metabolite), while an edge indicates a tentative link between two phenotypes (e.g., conversion between two metabolites).

10.1128/mBio.01470-21.5 FIG S4 The effect of sampling depth on IRCA. The quantitative relationship is shown between sampling depth and the observed cumulative IRCA traits (*cumPCC* and *cumAveDegree*) for an *in silico* population of 60 cells in the 7-day ramanome of C. reinhardtii CC124. (A to D) At each sampling depth, 100,000 permutations for *cumPCC* (938 cm^−1^ and 2,855 cm^−1^) and 10,000 permutations for *cumAveDegree* of sampling trials were performed, and the mean and increase rate of *cumPCC* (A and B) and *cumAveDegree* (C and D) were calculated. The minimal sampling depth, defined as the depth when no more than 1% of gain in cumulative IRCA traits is gained by sampling one more cell, is highlighted. The minimal sampling depth is high-lighted with red points and the corresponding numbers. Download FIG S4, TIF file, 0.4 MB. Copyright © 2021 He et al. 2021 He et al. https://creativecommons.org/licenses/by/4.0/ This content is distributed under the terms of the Creative Commons Attribution 4.0 International license.

An IRCN can reveal valuable features of the system. For example, in a 51-peak module of the CC124 N– 7-day IRCN (Fig. 4B; also see Fig. S5A), besides 478 cm^−1^ and 938 cm^−1^ for starch and 1,658 cm^−1^ and 2,853 cm^−1^ for TAG, many peaks of known or unknown assignments are present, suggesting additional between-metabolite conversions. In particular, 526 cm^−1^ (phosphatidylserine; PS) and 577 cm^−1^ (phosphatidylinositol; PI) exhibit strong NC with 968 cm^−1^, 1,302 cm^−1^, and 1,263 cm^−1^, which are all TAG markers (18) (Fig. 4B). As both PS and PI are main components of membrane lipids, these observations suggest the conversion between membrane lipids and TAG under N– at 7 days (37, 38). These findings are supported by (i) the concomitant TAG accumulation and membrane lipid degradation under stress for C. reinhardtii (39) and (ii) the stable, modest rise of cellular FAME (fatty acid methyl ester) levels yet dramatic fall of chloroplast membrane lipid level at the early stage of N– in C. reinhardtii (40). Notably, comparison of the 16 time series IRCNs of CC124 under N– revealed a deepening trend of the PS-TAG correlation and of the PI-TAG correlation (see Fig. S5B to E), suggesting the increasing extent of such conversions with time. Moreover, the NC between membrane lipids and TAG occurred as early as 8 h, much earlier than that between starch and TAG (Fig. 2O and P; 2 days), indicating that the membrane-lipid-TAG conversion precedes the starch-TAG conversion. On the other hand, in the N– 7-day IRCN, the starch-TAG NC is present, an indication of the starch-TAG conversion (Fig. 4B). Therefore, this local IRCN module detects multiple concomitant conversion processes that all produce TAG and reveals their dynamic features.

10.1128/mBio.01470-21.6 FIG S5 Correlation of the membrane-lipid and TAG contents among individual cells at each time point. (A) The mean SCRS of the CC124 7-day ramanome (as an example), showing the major peaks and their assignments. (B to E) The phenotypic correlation between membrane-lipid (*x*) and TAG (*y*) contents modeled by 526 cm^−1^ (phosphatidylserine or PS) and 1,263 cm^−1^ (TAG) are shown (B and C), as is that for 577 cm^−1^ (phosphatidylinositol or PI) and 1,263 cm^−1^ (D and E). In the scatterplots, each dot represents one cell. *ρ* is Pearson correlation coefficient among single cells (**, *P < *0.01; *, *P < *0.05), with the value indicating the mean of triplicates and the error bar indicating the standard deviation. The absence of correlation (*ρ* = 0) or presence of strong correlation (*ρ* ≥ 0.6 or *ρ* ≤ –0.6) is highlighted by red horizontal lines. Both PS and PI are the main components of membrane lipids. Download FIG S5, TIF file, 0.9 MB. Copyright © 2021 He et al. 2021 He et al. https://creativecommons.org/licenses/by/4.0/ This content is distributed under the terms of the Creative Commons Attribution 4.0 International license.

Notably, the IRCN can be readily expanded to include nodes representing those phenotypes that are underpinned by multiple Raman peaks. For example, the degree of unsaturation (DU), a key feature that determines the application and value of lipids, can be quantified by the ratio between the two Raman peaks of 1,658 cm^−1^ (unsaturated C=C bonds) and 1,441 cm^−1^ (saturated C-H_2_ bonds) (18, 41, 42). Inclusion of DU into the aforementioned time-series IRCNs revealed a local module of 17 peaks with functional assignment, where DU is highly negatively correlated with two starch peaks (938 cm^−1^ and 1,125 cm^−1^) and one protein peak (1,007 cm^−1^) at the late phase of N– (Fig. 4C; also see Fig. S6A and B; 2 days to 8 days). This suggests starch and proteins are converted to unsaturated lipids at the late phase of N–, consistent with the concordance between (i) degradation of proteins and starch and (ii) accumulation of unsaturated lipids (23, 28–32).

10.1128/mBio.01470-21.7 FIG S6 Local IRCNs that include the degree of lipid unsaturation (DU) and CD ratio. (A) A local IRCN that includes the DU of CC124 at 16 time points (threshold: strong negative correlation, *ρ* ≤ –0.6, *P < *0.05). (B) The temporal dynamics curves of correlations between DU and 17 characteristic Raman peaks. (C) A local IRCN that includes the CD ratio of CC124 under 25% D_2_O at 6 time points (*ρ* ≤ –0.6, *P < *0.05). (D) The temporal dynamics of correlations between CD ratio and 17 characteristic Raman peaks (plus DU). *ρ*, Pearson correlation coefficient among the phenotypes among single cells, with the value indicating the mean of triplicates and the error bar indicating the standard deviation. An absence of correlation (*ρ* = 0) or presence of strong correlation (*ρ* ≥ 0.6 or *ρ* ≤ –0.6) is highlighted by red horizontal lines. Download FIG S6, TIF file, 1.9 MB. Copyright © 2021 He et al. 2021 He et al. https://creativecommons.org/licenses/by/4.0/ This content is distributed under the terms of the Creative Commons Attribution 4.0 International license.

Moreover, IRCN can reveal the link between substrate intake and metabolite production of the cell. For example, cellular intake of D_2_O resulted in substitution of C-H bonds in intracellular macromolecules by C-D bonds. Therefore, the CD ratio, i.e., the ratio between the C-D bond area (2,040 to 2,300 cm^−1^) and the area of C-D plus C-H bonds (2,040 to 2,300 cm^−1^ and 2,800 to 3,050 cm^−1^), can measure cellular metabolic activity (43, 44). In a separate time-resolved experiment, D_2_O was fed to CC124 immediately after the removal of medium nitrogen (i.e., sampled for ramanomes in triplicates at 0 h, 12 h, 1 day, 2 days, 3 days, and 4 days under N–; see Fig. S6C; Materials and Methods). IRCA revealed that (i) specifically at 2 days, a module of 17 characteristic peaks was formed, in which the CD ratio exhibited strong NC (*ρ* ≤ –0.6, *P < *0.05) with 5 TAG peaks (1,263, 1,443, 1,656, 2,855, and 3,011 cm^−1^; Fig. 4D, also see Fig. S6C and D), suggesting that at 2 days, higher TAG-content cells exhibit lower D_2_O-assimilating activity (and visa versa). Thus, at 2 days, the algal population was at a most “diverse” metabolic state, where both low-TAG, active-“drinking” cells and high-TAG, inactive-drinking cells were abundant, while before 2 days the former dominated, and after 2 days the latter dominated. (ii) Between CD ratio and DU, no strong NC (*ρ* ≤ –0.6, *P < *0.05) was observed at any of the time points (Fig. 4D, also see Fig. S6C and D), except for weak correlation at 3 days (*ρ* = –0.50, *P < *0.01), suggesting that the DU of synthesized lipids increased with reducing C. reinhardtii vitality under the nitrogen-depletion stress (this finding is supported by GC-MS data [18]). (iii) In contrast to the TAG peaks, peaks of starch (the major carbon storage form of C. reinhardtii under N–) showed no correlation with CD ratio at any of the time points. Thus, starch synthesis appears to be mainly supported by endogenously derived H, while TAG synthesis requires exogenously supplied H. Therefore, IRCA is a new strategy to track the cellular destination of target substrate (in this case, the water).

Altogether, a choreography of interplay among water intake and major cellular products was revealed (Fig. 4E). (i) At 0 h, only the protein-starch conversion took place; (ii) at 12 h, both the protein-starch conversion and the D_2_O incorporation occurred; (iii) at 2 days, besides the protein-starch conversion, TAG became the most catabolically active component, and starch, PS, PI, and D_2_O all contributed to TAG synthesis; (iv) at 4 days, the three conversions of starch-TAG, PS-TAG, and PI-TAG still took place, but not the protein-starch and the D_2_O-TAG conversions. Therefore, IRCA can unveil metabolite conversions from a single snapshot of an isogenic population (i.e., one ramanome), while revealing the dynamics of such conversions via a temporal series of ramanomes.

### Global features of IRCNs reveal the degree of dynamics for metabolite conversions.

**FIG 5:**
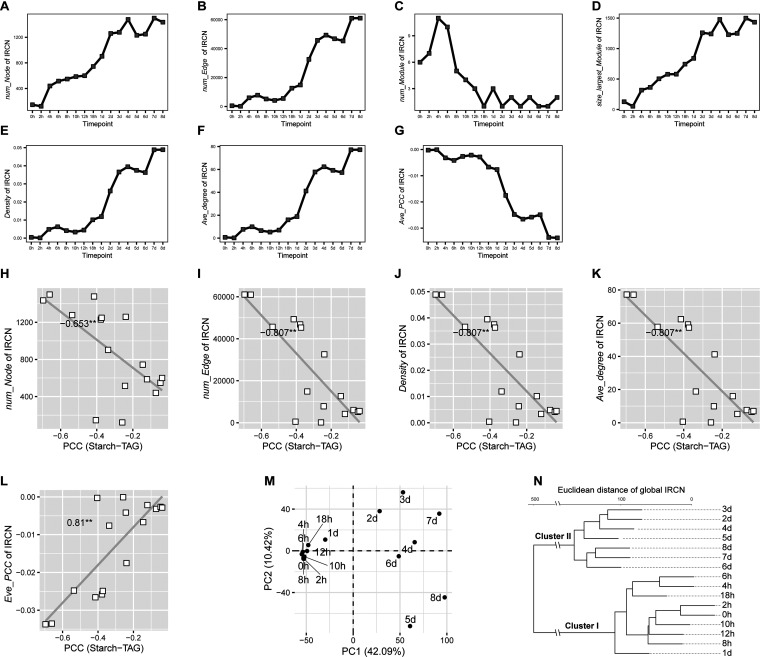
Intra-ramanome correlation network (IRCN) reveals global features of metabolite conversion dynamics. (A to G) Key network parameters derived from the time series IRCNs (*ρ* ≤ –0.6, *P < *0.05) of CC124 under N–, including number of nodes (A) (*num_Node*), number of edges (B) (*num_Edge*), number of modules (C) (*num_Module*; each module is a sub-IRCN not connected with any other nodes in the IRCN), size of the largest module (D) (*size_largest_Module*; number of nodes in the largest module of each IRCN), density (E) (*Density*, ratio of the number of edges divided by the number of all possible edges of the same nodes), average degree (F) (*ave_Degree*, average number of adjacent edges), and average PCC (G) (*ave_PCC*, sum of all significant strong negative correlations divided by all nodes). (H to L) Correlations between the starch-TAG conversion (i.e., PCC of starch-TAG) and key network parameters that include *num_Node* (H), *num_Edge* (I), *Density* (J), *ave_Degree* (K), and *ave_PCC* (L). (M and N) Also shown are clusters of IRCNs via PCA (M) and HCA (N) based on the correlation matrix of IRCN (strong negative correlations; *ρ* ≤ –0.6, *P < *0.05).

To probe the global features of IRCN, key network parameters were derived for the time series IRCNs of CC124 under N– (*ρ* ≤ –0.6, *P < *0.05; Fig. 5; heatmap of *ρ* shown in Fig. S7A). (i) The number of nodes (*num_Node*; Fig. 5A) increased (minimum [min] of 122 at 2 h and maximum [max] of 1,499 at 7 days), and so did the number of edges (*num_Edge*; *ρ* ≤ –0.6, *P < *0.05; Fig. 5B). (ii) The number of modules (*num_Module*; each module is a sub-IRCN that is not connected with any other nodes in the IRCN; Fig. 5C) decreased (max of 11 at 4 h and min of 1 at 18 h, 2 days, 4 days, 6 days, and 7 days). However, the size of the largest module (*size_largest_Module*; number of nodes in the largest module of each IRCN; Fig. 5D) increased greatly (min of 53 at 2 h and max of 1,499 at 7 days). (iii) Both the density (*Density*, number of edges divided by number of all possible edges for the same nodes; Fig. 5E) and the average degree (*ave_Degree*, average number of adjacent edges; Fig. 5F), both depicting the degree of network complexity, increased. In contrast, the average Pearson correlation coefficient (*ave_PCC*; Fig. 5G) became more negative (from –0.000078 at 2 h to –0.0335 at 7 days), indicating more frequent and more active conversions between the implicated metabolites.

10.1128/mBio.01470-21.8 FIG S7 The time-series IRCNs of CC124 under N– reveal the global features of metabolite conversion dynamics. (A) IRCNs (*ρ* ≤ –0.6, *P < *0.05) were shown as two-dimensional (2D) heatmaps. (B) Degree of the most active Raman peaks for the three largest modules in each IRCN (*ρ* ≤ –0.6, *P < *0.05). (C) The Δ*ρ* of the IRCNs (i.e., 0 h to 8 days). Δ*ρ* = *ρ*_max_ – *ρ*_min_. (D) The IRCNs of three ramanomes (CC4324_N–_72h, CC4333_N–_72h, and CC4565_N–_72h) in the 2D heatmap provide a global view of metabolite conversion modes of the various species. Mean, mean spectra of one ramanome; HI, the heterogeneity index of a Raman peak, which is defined as the RSD (relative standard deviation) of individual cells within a ramanome; degree, the degree for each peak in an IRCN. Download FIG S7, TIF file, 2.8 MB. Copyright © 2021 He et al. 2021 He et al. https://creativecommons.org/licenses/by/4.0/ This content is distributed under the terms of the Creative Commons Attribution 4.0 International license.

Across all time points, the dynamics of *num_Node*, *num_Edge*, *Density*, *ave_Degree*, and *ave_PCC* of 16 IRCNs were significantly correlated with the starch-TAG conversion (PCC of –0.653, –0.807, –0.807, –0.807. and 0.81, respectively; *P < *0.05; Fig. 5H to L). In addition, Raman peaks at the carbohydrate and lipid region were usually prominently found in the nodes with the highest degree (see Fig. S7B). Moreover, the 1,457 cm^−1^ region (the alkyl C–H_2_ bend of saturated lipids) dominated at the later phases under N– (i.e., 4 days, 5 days, 7 days, and 8 days), consistent with the turnover between lipid classes (saturated switching to unsaturated). Therefore, topological features of an IRCN can reveal both metabolically active compounds and their interconversions.

**FIG 6:**
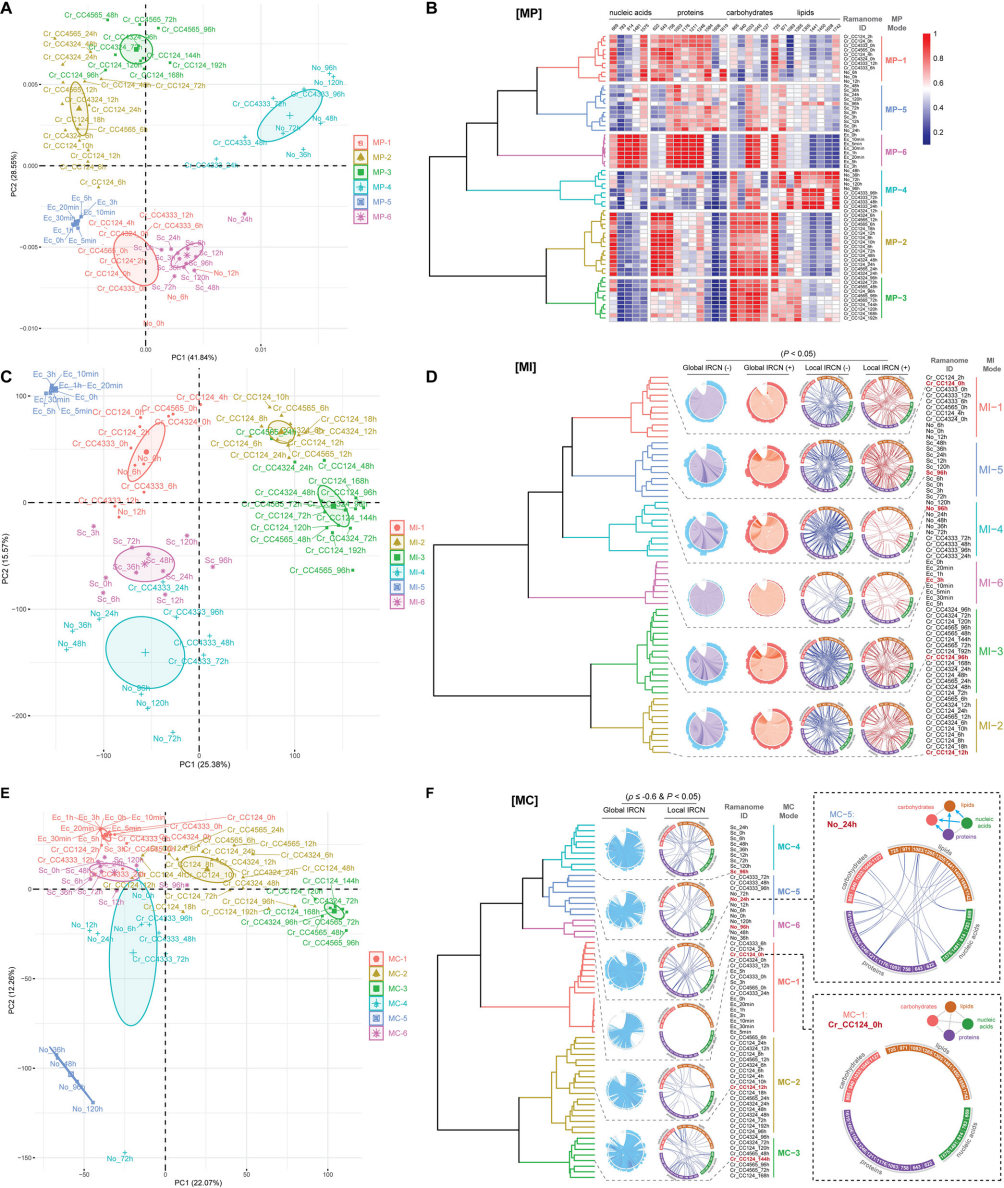
Clustering of ramanomes via the metabolite profiling (MP), metabolite interaction (MI), and metabolite conversion (MC) signatures. (A to F) The PCA and HCA clusters of 64 ramanomes based on MP (A and B) (mean SCRS), MI (C and D) (*P < *0.05), and MC (E and F) (*ρ* ≤ –0.6, *P < *0.05) are shown. Details for each of the Chlamydomonas reinhardtii populations (WT and starchless mutant series, C. reinhardtii [*Cr*]), *Nannochloropsis oceanica* (*No*), Saccharomyces cerevisiae (*Sc*), and Escherichia coli (*Ec*) are provided in Table S1. Details for the Raman barcodes and local IRCNs are also provided in Table S1. Global IRCNs were derived from Raman peaks from 600 cm^−1^ to 1,800 cm^−1^. Clusters are colored based on HCA.

Based on the matrix of *ρ* (*ρ* ≤ –0.6, *P < *0.05), the 16 CC124 IRCNs under N– can be classified as clusters I and II (Fig. 5M and 6N); I consists of those time points before 24 h, which are characterized by the global features of low *num_Edge* and low intensity of *ave_PCC*, and II includes those after 24 h, with global features of a sharp increase of *num_Edge* and of *ave_PCC*. Thus, 24 h is the temporal transition point (consistent with population-level measurement; see Fig. S1), when protein stopped degradation, starch stopped accumulation, and the early build-up of starch began to contribute to other cell activities, such as serving as the carbon source for lipid synthesis.

In addition, the global heatmap of Δ*ρ* (the difference between max *ρ* and min *ρ* in a ramanome series) reveals the dynamic change of the “hot spots” of metabolite conversion. For example, in the 16-time point CC124 N– process (see Fig. S7C), the Δ*ρ* between 1,441 cm^−1^ (alkyl C-H_2_ bend, CH_2_ scissoring, and CH_3_ bending; i.e., saturated lipids) and 1,402 cm^−1^ (bending modes of methyl groups; i.e., proteins) is one of the most prominent hot spots (1.18; highlighted in Fig. S7C), with a max *ρ* of 0.619 at 2 h (strong positive correlation) and min *ρ* of –0.557 at 7 days (strong NC). This suggests the temporal switch, from the synergic degradation of lipids and protein at 2 h to the protein-to-lipid conversion at 7 d, is a key dynamic feature of this process.

Furthermore, such heatmaps of *ρ* can measure the global similarity of metabolite conversion modes among ramanomes from different strains (see Fig. S7D). For example, at N– 72 h, comparison among IRCNs of CC4324 (wild-type), CC4333 (starchless mutant), and CC4565 (genetically complemented strain) reveal much higher similarity with CC4324 for CC4565 than for CC4333. Specifically, CC4333 is distinct due to the absence of several regions (dotted rectangle in Fig. S7D) found in both CC4324 and CC4565, such as (i) 867 to 970 cm^−1^ (conversion of starch to lipids) and (ii) 1,441 to 1,658 cm^−1^, and 1,441 to 1,744 cm^−1^ (conversions of saturated lipids to unsaturated lipids; solid rectangles in Fig. S7D). These IRCN-derived phenotypes of the three strains are consistent with their genotypes (Fig. 3).

### IRCN is a new “state”-specific metabolic signature of an isogenic population for diverse organisms.

A ramanome is essentially a single-cell-resolution metabolic snapshot of the cellular population (16, 21). To maximally extract the metabolic features from a ramanome (Fig. 1), three signatures are proposed, metabolite profile (MP; derived via the mean SCRS of a ramanome; Fig. 6A and B), metabolite interaction (MI; derived via the correlation matrix of a ramanome consisting of significant correlations of all pairwise Raman peaks [*P < *0.05]; Fig. 6C and D), and metabolite conversion (MC; derived via a correlation matrix of a ramanome that consists of strong NC of all pairwise Raman peaks [*ρ* ≤ 0.6, *P < *0.05]; Fig. 6E and F). To test their general applicability across organisms, MP, MI, and MC were derived for each of the 64 ramanomes from C. reinhardtii (wild type [WT] and the starchless mutant series), *N. oceanica*, Saccharomyces cerevisiae, and Escherichia coli (see Table S1; Materials and Methods). To facilitate interspecies comparison, the temporal ramanome data set of CC124 under N– along 16 time points was regarded as a standard “protein-starch-TAG” (PST) process, onto which the entire 64 ramanomes were “projected.”

Similarity-based clustering of MP reveals six modes (Fig. 6A and B). (i) MP-1 consists of PST 0 h, 2 h, and 4 h, plus the early phases under N– of C. reinhardtii and *N. oceanica*, indicative of high protein content but low content of starch or TAG. (ii) MP-2 includes PST 6 h, 8 h, 10 h, 12 h, 18 h, 24 h, 48 h, and 72 h, i.e., starch-producing C. reinhardtii strains (CC124, CC4324, and CC4565) at the middle phases (i.e., 6 h to 48 h) under N–, indicating high carbohydrate content. (iii) MP-3 includes PST 96 h, 120 h, 144 h, 168 h, and 192 h, i.e., mainly starch-producing C. reinhardtii strains (CC124, CC4324, and CC4565) at the later phase (i.e., 48 h to 192 h) under N–, indicating high carbohydrate content and the start of accumulating lipids (mainly neutral lipids such as TAG). (iv) MP-4 includes both the middle and the later phases of *N. oceanica* under N– (36 h, 48 h, 72 h, 96 h, and 120 h) and the later phases of starchless C. reinhardtii strains (CC4333) under N– (24 h, 48 h, 72 h, and 96 h), corresponding to high content of neutral lipids and low levels of nucleic acids, protein, and carbohydrates. (v) MP-5 consists of S. cerevisiae (0 h to 120 h), featuring high protein yet low carbohydrate and lipid amounts. (vi) MP-6 consists of E. coli under kanamycin (0 h to 5 h), featuring larger amounts of proteins and nucleic acid.

MI forms six clusters (Fig. 6C and D). (i) MI-1 consists of 0 h, 2 h, and 4 h of PST, i.e., the very early phases under N– of C. reinhardtii and *N. oceanica* strains, indicating that proteins began to degrade and were converted to other metabolites, such as starch. (ii) MI-2 consists of PST 6 h, 8 h, 10 h, 12 h, 18 h, and 24 h, i.e., the middle phase of starch-producing C. reinhardtii strains (CC124, CC4324, and CC4565) under N–, corresponding to high carbohydrate accumulation. (iii) MI-3 is composed of PST 48 h, 72 h, 96 h, 120 h, 144 h, 168 h, and 192 h, i.e., the later phase of starch-producing C. reinhardtii strains (CC124, CC4324, and CC4565) under N–, indicating the conversion of accumulated carbohydrates to lipids. (iv) MI-4 consists of both the middle and the later phases of *N. oceanica* under N– (24 h, 36 h, 48 h, 72 h, 96 h, and 120 h) and the later phase of the starchless CC4333 under N– (24 h, 48 h, 72 h, and 96 h), indicative of a state converting other metabolites such as nucleic acids, protein, and carbohydrates to lipids. (v and vi) S. cerevisiae (0 h to 120 h) and E. coli (0 h to 5 h) form MI-5 and MI-6, respectively, underscoring each species’ characteristic metabolite-interacting networks.

MC also formulates six modes (Fig. 6E and F). (i) MC-1 consists of 0 h and 2 h of PST, i.e., the early phase of C. reinhardtii strains under N– and all E. coli states—no or few strong pairwise NCs are found in these IRCNs. (ii) MC-2 consists of PST 4 h, 6 h, 8 h, 10 h, 12 h, 18 h, 24 h, 48 h, 72 h, and 96 h, plus the early and the middle stages of starch-producing C. reinhardtii mutants under N–, when proteins are converted into starch (see Fig. S1A). Notably, MC-2 showed more NC links between Raman peaks than MC-1, indicating more extensive metabolite conversions in MC-2. (iii) MC-3 is composed of PST 120 h, 144 h, and 168 h, i.e., the late stages of starch-producing C. reinhardtii strains under N–, when starch is converted into lipids (Fig. 2B). The NC links in IRCN of MC-3 are more abundant and stronger than those of MC-2, suggesting even more extensive and active metabolite conversions. (iv) MC-4 includes all the S. cerevisiae ramanome, which showed a distinct pattern of conversion among nucleic acids, proteins, carbohydrates, and lipids. (v) MC-5 mainly consists of the later phases of the starchless CC4333 and the early phase of *N. oceanica* strains under N–, corresponding to the metabolite-conversion pattern of lipid production. (vi) MC-6 mainly consists of the later phase of *N. oceanica* strains under N–, where proteins are converted into a large amount of lipids.

The MP, MI, and MC signatures, although inherently linked, can be highly distinct (see Fig. S8). Specifically, MP appears to show more species specificity, while MI and MC exhibit more state-specificity. For example, E. coli ramanomes are clustered with algae and yeast in MC-1; in contrast, E. coli ramanomes are solely clustered as MP-6. However, MC can be more sensitive in detecting cellular state change than MP and MI. For example, at 4 h under N–, the metabolite content of CC124 has yet to change (i.e., clustered with 0 h in MP-1; it did not switch to MP-2 until 6 h), yet the mode of metabolite conversion has already altered (i.e., clustered with 6 h to 96 h in MC-2).

10.1128/mBio.01470-21.9 FIG S8 Similarity and distinction among the clustering patterns based on MP, MI, or MC. Each node represents one ramanome. The same ramanomes found in MP, MI, or MC are linked by lines. The nodes and lines are colored based on strain. Download FIG S8, TIF file, 1.1 MB. Copyright © 2021 He et al. 2021 He et al. https://creativecommons.org/licenses/by/4.0/ This content is distributed under the terms of the Creative Commons Attribution 4.0 International license.

## DISCUSSION

Exploiting a fundamental and inherent property of all cellular systems, i.e., the heterogeneity in single-cell metabolism-related phenotypes (e.g., metabolite contents and substrate intake) among individual cells, here, we proposed and then biochemically and genetically validated the IRCA approach. IRCA is advantageous, as it unveils a comprehensive and landscape-like network of potential links among metabolic activities, in this case metabolite conversions or links between substrates and products, from a single instance of an isogenic population of living cells, rather than requiring a time or condition series of samples. This ability is of particular importance for those phenotyping experiments where spatiotemporal or condition-resolved sampling is a hurdle or a constraint.

Despite its need of just one snapshot of an isogenic population, an IRCN is information rich. From an IRCN, a large number of hypotheses on potential links among metabolism-related phenotypes can be generated simultaneously, based on the potentially millions of pairwise correlations from the Raman peaks that each potentially represents a phenotype or from the combinations of Raman peaks that model particular phenotypes, among the individual cells sampled for SCRS in a ramanome. The scope of such phenotypes is broad (16, 45), including but not limited to substrate intake (46), product synthesis (17, 18), and response to environmental stress (e.g., antimicrobial susceptibility [21, 44]). Based on the long and expanding list of such SCRS-derived phenotypes (16, 45), IRCNs can be constructed and then interpreted to mine the ramanome data space for new interphenotype links without *a priori* hypotheses. Moreover, since Raman microspectroscopy is label-free, noninvasive, and generally applicable to any cells, the strength of IRCA also includes high throughput, low cost, excellent scalability, and broad applicability. For example, acquisition of SCRS can be automated via flow-mode Raman cytometry or sorting (47, 48), suggesting the possibility of ultrafast, robust yet low-cost acquisition of ramanomes for IRCA. Therefore, IRCA presents a new dimension of “metabolism-related phenome” for cellular systems, which serves as a highly species- and state-specific signature of metabolic activity that captures not just the profile of Raman-sensitive metabolites (as well as other SCRS-derived phenotypes) but their dynamic links. This capacity of IRCA would enable a data-driven research strategy for profiling cellular metabolism.

On the other hand, the potential of IRCA is limited by its frequent inability to unambiguously assign Raman peaks to specific metabolites. For example, in this study, although ∼1,600 spectral resonance features were detected in each SCRS, only interconversion of macromolecules such as lipids, protein, and starch can be revealed, because a large number of vibrational features were shared among metabolites and cannot be assigned uniquely to individual metabolites. Even though the list of models that link spectral resonance features to a metabolite (or other metabolism-related phenotypes) has been rapidly growing (16), only a small portion of Raman peaks in an SCRS can be assigned to a defined metabolism-related phenotype at present. In addition, biological hypotheses for many of the edges, i.e., phenotype pairs showing significant NC, in an IRCN remain difficult to interpret or confirm. Therefore, new experimental and computational methods, such as stable-isotope probed SCRS (to track the assimilation of target substrate) and multivariate curve resolution-alternating least-squares (MCR) algorithms (to deconvolute macromolecular components from overlapping Raman peaks [49, 50]) should be developed to establish new assignments or to improve their specificity for the Raman peaks, so as to enrich and expand the actual information content of an IRCN.

Despite these present limitations, as demonstrated here using a number of microalgal, fungal, and bacterial species as examples, IRCA can serve as a valuable tool to rapidly discover features of metabolic dynamics of cellular systems, such as metabolite conversions. The capability of revealing such features from just one snapshot of an isogenic population has profound implications for designing phenotyping experiments.

## MATERIALS AND METHODS

### Strains and growth conditions.

For Chlamydomonas reinhardtii, a total of nine wild-type (WT), low-starch mutant and starchless mutant strains were employed (see Table S1). Specifically, CC124, CC406, and CC4324 were WT strains. The low-starch mutant CC4325 was derived by X-ray mutagenesis from CC124. The low-starch mutant CC4326 and the starchless mutants CC4333 and CC4334 were derived from CC4324 by random integration of cassette pARG7 into the nuclear genome (34, 35, 51). The starchless mutant CC4333 is deficient in the catalytic (small) subunit of ADP-glucose pyrophosphorylase, which interrupts synthesis of the ADP-glucose, a substrate for starch biosynthesis. The CC4334 mutant contains a disrupted isoamylase gene; thus, the level of starch is severely attenuated, but it accumulates a soluble glycogen-like product. CC4565 and CC4566 were genetically complementary strains of CC4333, derived by complementation with plasmid pSL18-STA6 (34). All these mutants can be obtained from the Chlamydomonas Resource Center (http://www.chlamycollection.org).

The C. reinhardtii cells were inoculated into the TAP (Tris acetate phosphate) liquid medium with or without arginine (100 μg ml^−1^) supplement under one-side continuous light (approximate 150 μmol photons m^−2^ s^−1^) at 25°C bubbled with air to ensure mixture and to prevent settling and were then grown to the late log phase in the N-replete TAP medium. Then they were reinoculated at 1 × 10^6^ cells/ml in parallel into the nitrogen-replete TAP medium (N+), the nitrogen-depleted TAP medium (N–; in which NH_4_Cl was omitted), or the 25% D_2_O nitrogen-depleted TAP medium (25% D_2_O N–), each in triplicates. Cultures at each of a series of time points were sampled in triplicate (see Table S1A).

For the *Nannochloropsis oceanica* IMET1 strain, cells were cultured in a modified f/2 liquid medium with 4 mM NO_3_^−^ under continuous light (approximately 50 μmol photons m^−2^ s^−1^) at 25°C and then induced in nitrogen-replete (N+) or nitrogen-depleted (N–) f/2 medium. The cultures were sampled at multiple time points of 0 h, 6 h, 12 h, 24 h, 48 h, 72 h, 96 h, and 120 h. For E. coli DH5α, cells were cultured in a glass tube with fresh LB medium (10g/liter NaCl, 10g/liter tryptone, 5g/liter yeast extract, 3.7 μg/ml kanamycin) at 37°C in a shaking incubator (130 rpm). Samples were collected at 0, 5, 10, 20, 30, 60, 180, and 300 min (21). For Saccharomyces cerevisiae Y50049, cells were cultured in a glass tube of fresh yeast extract-peptone-dextrose (YPD) medium at 30°C in a shaking incubator (200 rpm). Samples were collected at 0 h, 3 h, 6 h, 12 h, 24 h, 36 h, 48 h, 72 h, 96 h, and 120 h. All cultures and all sampling for further analysis were in triplicate.

### Acquisition of single-cell Raman spectra from an isogenic population of cells.

Raman spectra of individual cells were acquired using modified Raman microspectroscopy equipped with a confocal microscope with a ×50 PL magnifying dry objective (numerical aperture [NA] = 0.55, BX41; Olympus, UK) and a 532-nm Nd:YAG laser (Ventus, Laser Quantum Ltd., UK). The scattered photons were collected by a Newton electron multiplying charge coupled device (EMCCD) (Andor, UK) utilizing a 1,600 × 200 array of 16-μm pixels with thermoelectric cooling down to −70°C for negligible dark current. Before measurement, each sample was washed three times and resuspended in double-distilled water (ddH_2_O) to remove the culture medium. For algal and yeast samples, cells were loaded into a capillary tube (50-mm length by 1-mm width by 0.1-mm height; Camlab, UK) for SCRS acquisition. The power out of the objective was 100 mW. For each SCRS, the signal acquisition time was 2 s for C. reinhardtii and *N. oceanica* and 3 s for yeast. For C. reinhardtii and *N. oceanica*, prior to Raman signal acquisition, the cell was quenched with a 532-nm laser until the signal of fluorescent and resonantly enhanced biomolecules was no longer detectable. For each individual cell, a background spectrum was generated as the average of four spectra acquired from the liquid around the cell. For E. coli samples, cells were loaded onto a clean CaF_2_ slide and air dried before Raman measurement, and the SCRS of E. coli samples were collected as described (21).

Notably, a eukaryotic cell is generally larger than the laser focal spot obtained with a lens objective. In the case of a C. reinhardtii (∼10 μm in diameter), *N. oceanica* (2 to ∼3 μm in diameter), or S. cerevisiae (2 to ∼3 μm in diameter) cell, which was held in the single-beam gradient force trap under the aqueous conditions, the cell was rolling in random orientation when undergoing SCRS acquisition. Therefore, the SCRS acquired represents the overall metabolic state of the cell.

### Intra-ramanome correlation analysis.

The raw SCRS was first preprocessed with LabSpec 5 (Horiba Scientific, France), including background subtraction and baseline correction by a polynomial algorithm (with degree of 7). Then SCRS were normalized, followed by IRCA, which used a customized computational pipeline for data analysis and result visualization.

Due to potential technical variation (e.g., change of data collector, batch, etc.), SCRS from different ramanomes may have distinct spectral ranges and resolution. Therefore, prior to computing the MP signature and the MI and MC networks, the 64 ramanomes were standardized in three steps: (i) for spectral range, only the “fingerprint area” (600 to 1,800 cm^−1^) was extracted; (ii) spectral resolution was simulated to 1 cm^−1^, via the interpolation algorithm; (iii) spectral normalization was performed by division by its area.

For each IRCN, the correlation matrix of each ramanome was constructed by calculating the Pearson correlation coefficient (PCC; *ρ*) of all possible pairwise combinations of Raman peaks, among all the 60 cells sampled for the ramanome. Those pairs of Raman peaks with significant correlation were considered candidates that potentially indicate links between two metabolites (*P < *0.05), while those with strong negative correlations suggested potential conversions among two metabolites (*ρ* ≤ –0.6, *P < *0.05). The igraph package in R (http://www.r-project.org) was used to derive the key network properties and visualize the IRCNs (either from specific subsets of Raman peaks or from all of the Raman peaks). To probe the global features of an IRCN, key network parameters including number of nodes (*num_Node*), number of edges (*num_Edge*), number of modules (*num_Module*; each module is a sub-IRCN not connected with any other nodes in the IRCN), size of the largest module (*size_largest_Module*; number of nodes in the largest module of each IRCN), density (*Density*, ratio of the number of edges divided by the number of all possible edges of the same nodes) and average degree (*ave_Degree*, average number of adjacent edges), and average Pearson correlation coefficient (*ave_PCC*, sum of all significant strong negative correlations divided by all nodes), were derived. For global IRCNs, all Raman peaks were used. For simplified versions of IRCN that facilitate visualization, characteristic marker Raman peaks were used (669, 783, 814, and 1,481 cm^−1^ for nucleic acids; 622, 643, 758, 1,003, 1,176, 1,211, 1,246, 1,584, 1,606, and 1,619 cm^−1^ for proteins; 865, 940, 1,033, 1,045, and 1,127 cm^−1^ for carbohydrates; and 725, 971, 1,083, 1,265, 1,305, 1,441, 1,450, 1,658, and 1,742 cm^−1^ for lipids; see Table S1B).

To characterize, compare, and cluster ramanomes, the three signatures of MP, MI, and MC were proposed. For MP, the mean SCRS of each ramanome was used. The MI network of a ramanome was generated as follows: (i) a correlation matrix was constructed by calculating the PCC of all possible pairwise Raman peaks; (ii) significant correlations (*P < *0.05) were tabulated, while those with no significant difference were counted as 0 (no correlation). The MC network of a ramanome was generated by including only those significant, strongly negative correlations (*ρ* ≤ –0.6, *P < *0.05). To measure the similarity, pairwise Euclidean distances were calculated. Then hierarchical cluster analysis (HCA) was performed with Ward’s algorithm, and six clusters were produced (52, 53). Principal-component analysis (PCA; the factoextra package in R) was used to visualize the MP, MI, and MC signatures.

### Data availability.

The data that support the findings of this study are available from the corresponding author, Jian Xu, upon reasonable request.

## References

[1] Castrillo JI, Pir P, Oliver SG. 2013. Yeast systems biology: towards a systems understanding of regulation of eukaryotic networks in complex diseases and biotechnology, p 343–365. *In* Walhout AJM, Vidal M, Dekker J (ed), Handbook of systems biology. Academic Press, San Diego, CA. doi:10.1016/B978-0-12-385944-0.00018-6.

[2] Wang M, Carver JJ, Phelan VV, Sanchez LM, Garg N, Peng Y, Nguyen DD, Watrous J, Kapono CA, Luzzatto-Knaan T, Porto C, Bouslimani A, Melnik AV, Meehan MJ, Liu W-T, Crüsemann M, Boudreau PD, Esquenazi E, Sandoval-Calderón M, Kersten RD, Pace LA, Quinn RA, Duncan KR, Hsu C-C, Floros DJ, Gavilan RG, Kleigrewe K, Northen T, Dutton RJ, Parrot D, Carlson EE, Aigle B, Michelsen CF, Jelsbak L, Sohlenkamp C, Pevzner P, Edlund A, McLean J, Piel J, Murphy BT, Gerwick L, Liaw C-C, Yang Y-L, Humpf H-U, Maansson M, Keyzers RA, Sims AC, Johnson AR, Sidebottom AM, Sedio BE, et al. 2016. Sharing and community curation of mass spectrometry data with global natural products social molecular networking. Nat Biotechnol 34:828–837. doi:10.1038/nbt.3597. 27504778 PMC5321674

[3] Power RA, Parkhill J, de Oliveira T. 2017. Microbial genome-wide association studies: lessons from human GWAS. Nat Rev Genet 18:41–50. doi:10.1038/nrg.2016.132. 27840430

[4] Nicholson JK, Holmes E, Elliott P. 2008. The metabolome-wide association study: a new look at human disease risk factors. J Proteome Res 7:3637–3638. doi:10.1021/pr8005099. 18707153

[5] Go Y, Walker DI, Soltow QA, Uppal K, Wachtman LM, Strobel FH, Pennell K, Promislow DEL, Jones DP. 2015. Metabolome-wide association study of phenylalanine in plasma of common marmosets. Amino Acids 47:589–601. doi:10.1007/s00726-014-1893-x. 25526869 PMC4329081

[6] Uppal K, Soltow QA, Promislow DEL, Wachtman LM, Quyyumi AA, Jones DP. 2015. MetabNet: an R package for metabolic association analysis of high-resolution metabolomics data. Front Bioeng Biotech 3:87. doi:10.3389/fbioe.2015.00087.. PMC4464066 26125020

[7] Beck T, Shorter T, Brookes AJ. 2020. GWAS Central: a comprehensive resource for the discovery and comparison of genotype and phenotype data from genome-wide association studies. Nucleic Acids Res 48:933–940. doi:10.1093/nar/gkz895.. PMC7145571 31612961

[8] Nishino J, Ochi H, Kochi Y, Tsunoda T, Matsui S. 2018. Sample size for successful genome-wide association study of major depressive disorder. Front Genet 9:227. doi:10.3389/fgene.2018.00227. 30002671 PMC6032046

[9] Suarez-Diez M, Saccenti E. 2015. Effects of sample size and dimensionality on the performance of four algorithms for inference of association networks in metabonomics. J Proteome Res 14:5119–5130. doi:10.1021/acs.jproteome.5b00344. 26496246

[10] Li J, Han D, Wang D, Ning K, Jia J, Wei L, Jing X, Huang S, Chen J, Li Y, Hu Q, Xu J. 2014. Choreography of transcriptomes and lipidomes of Nannochloropsis reveals the mechanisms of oil synthesis in microalgae. Plant Cell 26:1645–1665. doi:10.1105/tpc.113.121418. 24692423 PMC4036577

[11] Sanchez A, Golding I. 2013. Genetic determinants and cellular constraints in noisy gene expression. Science 342:1188–1193. doi:10.1126/science.1242975. 24311680 PMC4045091

[12] Ray LB. 2013. Cells go solo. Science 342:1187–1187. doi:10.1126/science.342.6163.1187. 24311679

[13] Notingher I, Hench LL. 2006. Raman microspectroscopy: a noninvasive tool for studies of individual living cells in vitro. Expert Rev Med Devices 3:215–234. doi:10.1586/17434440.3.2.215. 16515388

[14] Noothalapati Venkata Hemanth N, Shigeto S. 2012. Stable isotope-labeled Raman imaging reveals dynamic proteome localization to lipid droplets in single fission yeast cells. Chem Biol 19:1373–1380. doi:10.1016/j.chembiol.2012.08.020. 23177192

[15] Huang WE, Griffiths RI, Thompson IP, Bailey MJ, Whiteley AS. 2004. Raman microscopic analysis of single microbial cells. Anal Chem 76:4452–4458. doi:10.1021/ac049753k. 15283587

[16] He Y, Wang X, Ma B, Xu J. 2019. Ramanome technology platform for label-free screening and sorting of microbial cell factories at single-cell resolution. Biotechnol Adv 37:107388. doi:10.1016/j.biotechadv.2019.04.010. 31152870

[17] Ji Y, He Y, Cui Y, Wang T, Wang Y, Li Y, Huang WE, Xu J. 2014. Raman spectroscopy provides a rapid, non‐invasive method for quantitation of starch in live, unicellular microalgae. Biotechnol J 9:1512–1518. doi:10.1002/biot.201400165. 24906189

[18] He Y, Zhang P, Huang S, Wang T, Ji Y, Xu J. 2017. Label-free, simultaneous quantification of starch, protein and triacylglycerol in single microalgal cells. Biotechnol Biofuels 10:275–292. doi:10.1186/s13068-017-0967-x. 29177009 PMC5693592

[19] Brackmann C, Norbeck J, Åkeson M, Bosch D, Larsson C, Gustafsson L, Enejder A. 2009. CARS microscopy of lipid stores in yeast: the impact of nutritional state and genetic background. J Raman Spectrosc 40:748–756. doi:10.1002/jrs.2356.

[20] Collins AM, Jones HDT, Danxiang H, Qiang H, Beechem TE, Timlin JA. 2011. Carotenoid distribution in living cells of Haematococcus pluvialis (Chlorophyceae). PLoS One 6:e24302. doi:10.1371/journal.pone.0024302. 21915307 PMC3167842

[21] Teng L, Wang X, Xiaojun W, Honglei G, Lihui R, Tingting W, Wang Y, Yuetong J, Wei E, Huang, Xu J. 2016. Label-free, rapid and quantitative phenotyping of stress response in E. coli via ramanome. Sci Rep 6:34359. doi:10.1038/srep34359. 27756907 PMC5069462

[22] Msanne J, Xu D, Konda AR, Casas M, Awada T, Cahoon EB, Cerutti H. 2012. Metabolic and gene expression changes triggered by nitrogen deprivation in the photoautotrophically grown microalgae Chlamydomonas reinhardtii and Coccomyxa sp. C-169. Phytochemistry 75:50–59. doi:10.1016/j.phytochem.2011.12.007. 22226037

[23] Fan J, Yan C, Andre C, Shanklin J, Schwender J, Xu C. 2012. Oil accumulation is controlled by carbon precursor supply for fatty acid synthesis in Chlamydomonas reinhardtii. Plant Cell Physiol 53:1380–1390. doi:10.1093/pcp/pcs082. 22642988

[24] Li Y, Han D, Hu G, Dauvillee D, Sommerfeld M, Ball S, Hu Q. 2010. Chlamydomonas starchless mutant defective in ADP-glucose pyrophosphorylase hyper-accumulates triacylglycerol. Metab Eng 12:387–391. doi:10.1016/j.ymben.2010.02.002. 20172043

[25] Goodenough U, Blaby I, Casero D, Gallaher SD, Goodson C, Johnson S, Lee J, Merchant SS, Pellegrini M, Roth R, Rusch J, Singh M, Umen JG, Weiss TL, Wulan T. 2014. The path to triacylglyceride obesity in the sta6 strain of Chlamydomonas reinhardtii. Eukaryot Cell 13:591–613. doi:10.1128/EC.00013-14. 24585881 PMC4060482

[26] Work VH, Radakovits R, Jinkerson RE, Meuser JE, Elliott LG, Vinyard DJ, Laurens LML, Dismukes GC, Posewitz MC. 2010. Increased lipid accumulation in the Chlamydomonas reinhardtii sta7-10 starchless isoamylase mutant and increased carbohydrate synthesis in complemented strains. Eukaryot Cell 9:1251–1261. doi:10.1128/EC.00075-10. 20562225 PMC2918938

[27] Johnson X, Alric J. 2013. Central carbon metabolism and electron transport in Chlamydomonas reinhardtii: metabolic constraints for carbon partitioning between oil and starch. Eukaryot Cell 12:776–793. doi:10.1128/EC.00318-12. 23543671 PMC3675994

[28] Fan J, Ning K, Zeng X, Luo Y, Wang D, Hu J, Li J, Xu H, Huang J, Wan M, Wang W, Zhang D, Shen G, Run C, Liao J, Fang L, Huang S, Jing X, Su X, Wang A, Bai L, Hu Z, Xu J, Li Y. 2015. Genomic foundation of starch-to-lipid switch in oleaginous Chlorella spp. Plant Physiol 169:2444–2461. doi:10.1104/pp.15.01174. 26486592 PMC4677908

[29] Vigeolas H, Möhlmann T, Martini N, Neuhaus HE, Geigenberger P. 2004. Embryo-specific reduction of ADP-Glc pyrophosphorylase leads to an inhibition of starch synthesis and a delay in oil accumulation in developing seeds of oilseed rape. Plant Physiol 136:2676–2686. doi:10.1104/pp.104.046854. 15333758 PMC523332

[30] Andriotis VME, Pike MJ, Kular B, Rawsthorne S, Smith AM. 2010. Starch turnover in developing oilseed embryos. New Phytol 187:791–804. doi:10.1111/j.1469-8137.2010.03311.x. 20546137

[31] Heredia-Arroyo T, Wei W, Hu B. 2010. Oil accumulation via heterotrophic/mixotrophic Chlorella protothecoides. Appl Biochem Biotechnol 162:1978–1995. doi:10.1007/s12010-010-8974-4. 20443076

[32] Wei X, Liu L, Chao W, Chen Y, Wu Q. 2010. 13C-tracer and gas chromatography-mass spectrometry analyses reveal metabolic flux distribution in the oleaginous microalga Chlorella protothecoides. Plant Physiol 154:1001–1011. doi:10.1104/pp.110.158956. 20720172 PMC2948989

[33] Ball S, Marianne T, Dirick L, Fresnoy M, Delrue B, Decq A. 1991. A Chlamydomonas reinhardtii low-starch mutant is defective for 3-phosphoglycerate activation and orthophosphate inhibition of ADP-glucose pyrophosphorylase. Planta 185:17–26. doi:10.1007/BF00194509. 24186274

[34] Siaut M, Cuiné S, Cagnon C, Fessler B, Nguyen M, Carrier P, Beyly A, Beisson F, Triantaphylidès C, Li-Beisson Y, Peltier G. 2011. Oil accumulation in the model green alga Chlamydomonas reinhardtii: characterization, variability between common laboratory strains and relationship with starch reserves. BMC Biotechnol 11:7. doi:10.1186/1472-6750-11-7. 21255402 PMC3036615

[35] Zabawinski C, Van den Koornhuyse N, D’Hulst C, Schlichting R, Giersch C, Delrue B, Lacroix J, Preiss J, Ball S. 2001. Starchless mutants of Chlamydomonas reinhardtii lack the small subunit of a heterotetrameric ADP-glucose pyrophosphorylase. J Bacteriol 183:1069–1077. doi:10.1128/JB.183.3.1069-1077.2001. 11208806 PMC94975

[36] Mouille G, Maddelein M, Libessart N, Talaga P, Decq A, Delrue B, Ball S. 1996. Preamylopectin processing: a mandatory step for starch biosynthesis in plants. Plant Cell 8:1353–1366. doi:10.1105/tpc.8.8.1353. 12239416 PMC161254

[37] Krafft C, Neudert L, Simat T, Salzer R. 2005. Near infrared Raman spectra of human brain lipids. Spectrochim Acta A Mol Biomol Spectrosc 61:1529–1535. doi:10.1016/j.saa.2004.11.017. 15820887

[38] Movasaghi Z, Rehman S, Rehman IU. 2007. Raman spectroscopy of biological tissues. Appl Spectrosc Rev 42:493–541. doi:10.1080/05704920701551530.

[39] Yoon K, Han D, Li Y, Sommerfeld M, Hu Q. 2012. Phospholipid:diacylglycerol acyltransferase is a multifunctional enzyme involved in membrane lipid turnover and degradation while synthesizing triacylglycerol in the unicellular green microalga Chlamydomonas reinhardtii. Plant Cell 24:3708–3724. doi:10.1105/tpc.112.100701. 23012436 PMC3480297

[40] Juergens MT, Disbrow B, Shachar-Hill Y. 2016. The relationship of triacylglycerol and starch accumulation to carbon and energy flows during nutrient deprivation in Chlamydomonas. Plant Physiol 171:2445–2457. doi:10.1104/pp.16.00761. 27325664 PMC4972295

[41] Ota S, Alexandr J, Zdeněk P, Pavel Z, Ladislav N, Jan T, Petr K, Martin T. 2010. Raman microspectroscopy of individual algal cells: sensing unsaturation of storage lipids in vivo. Sensors 10:8635–8651. doi:10.3390/s100908635. 22163676 PMC3231231

[42] Wu H, Volponi JV, Oliver AE, Parikh AN, Simmons BA, Seema S. 2011. In vivo lipidomics using single-cell Raman spectroscopy. Proc Natl Acad Sci USA 108:3809–3814. doi:10.1073/pnas.1009043108. 21310969 PMC3048102

[43] Berry D, Mader E, Lee TK, Woebken D, Wang Y, Zhu D, Palatinszky M, Schintlmeister A, Schmid MC, Hanson BT. 2014. Tracking heavy water (D2O) incorporation for identifying and sorting active microbial cells. P Natl Acad Sci USA 112:194–203. doi:10.1073/pnas.1420406112. PMC4299247 25550518

[44] Tao Y, Wang Y, Huang S, Zhu P, Huang WE, Ling JQ, Xu J. 2017. Metabolic-activity based assessment of antimicrobial effects by D2O-labeled single-cell Raman microspectroscopy. Anal Chem 89:4108–4115. doi:10.1021/acs.analchem.6b05051. 28282113

[45] Wang Y, Song Y, Tao Y, Muhamadali H, Goodacre R, Zhou N, Preston GM, Xu J, Huang WE. 2016. Reverse and multiple stable isotope probing to study bacterial metabolism and interactions at the single cell level. Anal Chem 88:9443–9450. doi:10.1021/acs.analchem.6b01602. 27588325

[46] Hatzenpichler R, Krukenberg V, Spietz R, Jay Z. 2020. Next-generation physiology approaches to study microbiome function at single cell level. Nat Rev Microbiol 18:241–256. doi:10.1038/s41579-020-0323-1. 32055027 PMC7133793

[47] Hiramatsu K, Ideguchi T, Yonamine Y, Lee S, Luo Y, Hashimoto K, Ito T, Hase M, Park JW, Kasai Y, Sakuma S, Hayakawa T, Arai F, Hoshino Y, Goda K. 2019. High-throughput label-free molecular fingerprinting flow cytometry. Sci Adv 5:eaau0241. doi:10.1126/sciadv.aau0241. 30746443 PMC6357763

[48] Wang X, Xin Y, Ren L, Sun Z, Zhu P, Ji Y, Li C, Xu J, Ma B. 2020. Positive dielectrophoresis-based Raman-activated droplet sorting for culture-free and label-free screening of enzyme function in vivo. Sci Adv 6:eabb3521. doi:10.1126/sciadv.abb3521. 32821836 PMC7413728

[49] Ando M, Hamaguchi H. 2014. Molecular component distribution imaging of living cells by multivariate curve resolution analysis of space-resolved Raman spectra. J Biomed Opt 19:e011016. doi:10.1117/1.JBO.19.1.011016. 24108582

[50] Noothalapati H, Iwasaki K, Yamamoto T. 2017. Biological and medical applications of multivariate curve resolution assisted Raman spectroscopy. Anal Sci 33:15–22. doi:10.2116/analsci.33.15. 28070069

[51] Li Y, Han D, Hu G, Sommerfeld M, Hu Q. 2010. Inhibition of starch synthesis results in overproduction of lipids in Chlamydomonas reinhardtii. Biotechnol Bioeng 107:258–268. doi:10.1002/bit.22807. 20506159

[52] Lu W, Chen X, Wang L, Li H, Fu YV. 2020. Combination of an artificial intelligence approach and laser tweezers Raman spectroscopy for microbial identification. Anal Chem 92:6288–6296. doi:10.1021/acs.analchem.9b04946. 32281780

[53] Choo-Smith LP, Maquelin K, van Vreeswijk T, Bruining HA, Puppels GJ, Ngo Thi NA, Kirschner C, Naumann D, Ami D, Villa AM, Orsini F, Doglia SM, Lamfarraj H, Sockalingum GD, Manfait M, Allouch P, Endtz HP. 2001. Investigating microbial (micro)colony heterogeneity by vibrational spectroscopy. Appl Environ Microbiol 67:1461–1469. doi:10.1128/AEM.67.4.1461-1469.2001. 11282591 PMC92755

