# Supplementary material for: Intra-Ramanome Correlation Analysis Unveils Metabolite Conversion Network from an Isogenic Population of Cells
Source: mBio. 2021 Aug 31;12(4):e01470-21. doi: 10.1128/mBio.01470-21 (PMC8406334; doi:10.1128/mBio.01470-21)
Supplement: TEXT S1 [file mbio.01470-21-s0001.doc]

**Text S1**

**Intra-Ramanome Correlation Analysis Unveils Metabolite Conversion Network from an Isogenic Population of Cells**

Yuehui He^1,2,3^, Shi Huang^1,2,3,4^, Peng Zhang^1,2,3^, Yuetong Ji^1,2,3^, Jian Xu^1,2,3*^

^1^Single-Cell Center, CAS Key Laboratory of Biofuels, Shandong Key Laboratory of Energy Genetics and Shandong Institute of Energy Research, Qingdao Institute of BioEnergy and Bioprocess Technology, Chinese Academy of Sciences, Qingdao, Shandong, China

^2^Laboratory for Marine Biology and Biotechnology, Qingdao National Laboratory for Marine Science and Technology, Qingdao, Shandong, China

^3^University of Chinese Academy of Sciences, Beijing, China

^4^Department of Pediatrics and Center for Microbiome Innovation at Jacobs School of Engineering, University of California San Diego, La Jolla, CA 92093, USA

^*^Corresponding to: Jian Xu (xujian@qibebt.ac.cn), Phone: (86) 532-80662653, Fax: (86) 532-80662654

**Supplemental Materials and Methods**

**Quantification of starch, protein and TAG contents (at the population level) for the *Chlamydomonas reinhardtii* wild-type strain CC124**

Dry algal biomass was obtained via lyophilization by a vacuum freezing dryer. The starch content of dry algal biomass was quantified using an enzymatic starch assay kit (Amyloglucosidase/α-amylase Method; K-TSTA 07/11; Megazyme, Ireland). Briefly, samples of about 20mg dry algal power were treated with 80% ethanol to remove sugars, hydrolyzed into soluble maltodextrins with thermostable α-amylase and then digested into D-glucose with amyloglucosidase. The glucose produced was treated with a reagent containing glucose oxidase, peroxidase and 4-aminoantipyrine and then quantified spectrophotometrically at 510 nm wavelength.

The total protein content of algal cultures was measured as previously reported (1). Briefly, ~ 10 mg of lyophilized algal biomass was hydrolyzed in 200 μL lysis buffer (1 M sodium hydroxide, NaOH) and then incubated at 80 °C for 10 min by water bath. Then 800 μL ddH_2_O was added to the hydrolysate to bring the volume to 1 mL. Cellular debris was centrifuged at 12,000 g for 30 min before the supernatant was transferred to a new tube. The extraction was repeated two more times and all the supernatant extracts were pooled together. Then total protein in the supernatant was determined by the BCA Protein Assay kit (cw0014s; CWBio, China) via the manufacturer’s protocol.

To analyze the contents of total lipids and TAG in an algal cell population, GC-MS and TLC-GC-MS were performed (1). The procedures mainly include total lipid extraction, TLC, and profiling of FAMEs. Briefly, total lipids of ~30mg lyophilized algal powder were extracted with 6mL chloroform:methanol (2:1, v/v) and recovered in chloroform. For TAG quantification, ~ 0.5μg of lipid extract was loaded onto 10 × 20cm silica gel 60 (Merck KGaA, Darmstadt, Germany) TLC plates. TAG was separated, visualized and scraped from the plate, and then extracted with chloroform:methanol (2:1, v/v) from the TLC powder. The FAMEs were derived from all of the TAG extracts by acid-catalyzed transmethylation and then ~1mg lipid extracts were analyzed on an Agilent 7890-5975C gas chromatography mass spectrometer fitted with a HP-INNOWAX 30m × 0.25mm × 0.25μm column. The FAMEs were quantified using pentadecane as the internal standard and C8-C24 FAMEs mix as FAMEs standards.

**Quantifying the influence of sampling depth on Intra-Ramanome Correlation Analysis**

To assess the influence of sampling depth on IRCA, we first defined two parameters for an IRCN: (*i*) the cumulative mean of Pearson correlation coefficient (i.e., “*cumPCC*”) between pairwise Raman peaks, and (*ii*) the average degree of the IRCN (i.e., “*cumAveDegree*”). For a given ramanome, we randomly sampled the SCRS at a particular sampling depth which ranges from three to 60 cells. At each sampling depth, the trials were performed for 100000 times for *cumPCC* (10000 times for *cumAveDegree*). For each trial, *cumPCC* and *cumAveDegree* were calculated. The two parameters were then respectively plotted against the sampling depth, so as to quantitatively assess how the choice of sampling depth affects the accuracy and reliability in measuring these features.

For a ramanome, to determine whether the IRCA parameters of *cumPCC* and *cumAveDegree* are saturated at a certain sampling depth, we defined the “rate of *cumTrait*” (where “*cumTrait*” is either *cumPCC* or *cumAveDegree*) (2):


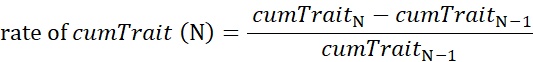


N: Sample Depth; *cumTrait*_N_: *cumTrait* at the sampling depth of N cells for a population; *cumTrait*_N-1_: *cumTrait* at the sampling depth of (N-1) cells for a population. The relationship between the rate of *cumTrait* and sampling depth was thus plotted. We set a cutoff of 1% for the rate of *cumTrait* to define the “minimal sampling depth”, which means, at this particular sampling depth, no more than 1% of gain in *cumTrait* is allowed by sampling one more cell.

**Supplemental Results**

**The effect of sampling depth on Intra-Ramanome Correlation Analysis**

To provide a rational basis for determining the proper sampling depth for IRCA, we tested the link between sampling depth and (*i*) the Pearson correlation coefficient (PCC) of pairwise Raman peaks, and (*ii*) the average degree of the IRCN (see **Supplemental Materials and Methods**). The cumulative PCC of pairwise Raman peaks (*cumPCC*) and cumulative average degree of an IRCN (*cumAveDegree*) were derived from each of the *in silico* trials of sub-sets of SCRS randomly sampled from the full ramanome at a particular sampling depth. The parameter of “minimal sampling depth” is thus designated as the sampling depth at a cutoff of 1% for the *cumTrait* (either *cumPCC* or *cumAveDegree*), i.e., at this particular sampling depth, no more than 1% of increase in *cumTrait* will be gained by sampling one more cell (see **Supplemental Materials and Methods**).

The minimal sampling depth varied with both the Raman peaks and the timepoints. For PCC of Raman peaks of 938 cm^-1^ (a starch-related peak) and 2855 cm^-1^ (a TAG-related peak), they vary from 4 (12h) to 10 (4h). For example, for the 60-cell collection at 7d, the “Minimal Sampling Depth” is 5 cells (see **Fig. S4A, B**), which is much lower than our actual sample depth (i.e., totally 60 cells for each time point under each condition). For the calculation of the average Degree (i.e., a key parameter for IRCN) of the 60-cell collection at 7d, the “Minimal Sampling Depth” is 33 cells (see **Fig. S4C, D**), which is also lower than the actual sample depth at each of the time points (i.e., 60 cells). Therefore, the sampling depth of 60 cells for each ramanome satisfied the construction of an IRCN.

**Abbreviations**

*ave_Degree*: Average degree (average number of adjacent edges)

*ave_PCC: Average* PCC (sum of all significant strong negative correlation divided by all nodes)

CD ratio: The ratio between C-D bond area (2040 to 2300 cm^-1^) and area of C-D plus C-H bonds (2040 to 2300 cm^-1^ and 2800 to 3050 cm^-1^)

*Cr*: *Chlamydomonas reinhardtii*

*cumAveDegree*: The cumulative average degree of the IRCN

*cumPCC*: The cumulative mean of Pearson correlation coefficient

*Density*: Density (ratio of the number of edges divided by the number of all possible edges of the same nodes)

*Ec*: *Escherichia coli*

FAMEs: Fatty acid methyl esters

HCA: Hierarchical cluster analysis

IRCA: Intra-Ramanome Correlation Analysis

IRCN: Intra-Ramanome Correlation Network

MC: Metabolite conversion

MI: Metabolite interaction

MP: Metabolite profile

N-: Nitrogen-depleted TAP medium

N+: Nitrogen-replete TAP medium

NC: Negative correlation

*No*: *Nannochloropsis oceanica*

*num_Edge*: Number of edges

*num_Module*: Number of modules (each module is a sub-IRCN not connected with any other nodes in the IRCN)

*num_Node*: Number of nodes

PC: Positive correlation

PCA: Principal component analysis

PI: Phosphatidylinositol

PS: Phosphatidylserine

PST: the Protein-Starch-TAG process

R^2^: Coefficient value

*Sc*: *Saccharomyces cerevisiae*

SCRS: Single-cell Raman Spectra

*size_largest_Module*: Size of the largest module (number of nodes in the largest module of each IRCN),

TAG: Triacylglycerol

TAP: Tris acetate phosphate

WT: Wild-type strain

Δ*ρ*: The difference between max ρ and min ρ in a ramanome series

*ρ*: Pearson correlation coefficient

**References**

1. Jia J, Han D, Gerken HG, Li Y, Sommerfeld M, Hu Q, Xu J. 2015. Molecular mechanisms for photosynthetic carbon partitioning into storage neutral lipids in Nannochloropsis oceanica under nitrogen-depletion conditions. Algal Res 7:66-77. https://doi.org/10.1016/j.algal.2014.11.005.

2. He Y, Zhang P, Huang S, Wang T, Ji Y, Xu J. 2017. Label-free, simultaneous quantification of starch, protein and triacylglycerol in single microalgal cells. Biotechnol Biofuels 10:275-292. https://doi.org/10.1186/s13068-017-0967-x.
