# Supplementary material for: Intra-Ramanome Correlation Analysis Unveils Metabolite Conversion Network from an Isogenic Population of Cells
Source: mBio. 2021 Aug 31;12(4):e01470-21. doi: 10.1128/mBio.01470-21 (PMC8406334; doi:10.1128/mBio.01470-21)
Supplement: Table S1 [file mbio.01470-21-st001.doc]

**Table S1A**. Microalgal and microbial strains, culture conditions and sampled time points for IRCA in this study.

| **Species** | **Strains** | **Condition** | **Timepoint** | **Description** |
| --- | --- | --- | --- | --- |
| *Chlamydomonas reinhardtii* | CC124 | TAP, N- | 0h, 2h, 4h, 6h, 8h, 10h, 12h, 18h, 24h, 48h, 72h, 96h, 120h, 144h, 168h, 192h | Wild-type |
|  | CC124 | TAP, N- | 0h, 6h, 12h, 24h, 48h, 72h, 96h | Wild-type |
|  | CC4325 | TAP, N- | 0h, 6h, 12h, 24h, 48h, 72h, 96h | Low-starch mutant |
|  | CC406 | TAP, N- | 0h, 6h, 12h, 24h, 48h, 72h, 96h | Wild-type, cell wall deficient |
|  | CC4324 | TAP, N- | 0h, 6h, 12h, 24h, 48h, 72h, 96h | Wild-type, cell wall deficient |
|  | CC4326 | TAP, N- | 0h, 6h, 12h, 24h, 48h, 72h, 96h | Low-starch mutant |
|  | CC4333 | TAP, N- | 0h, 6h, 12h, 24h, 48h, 72h, 96h | Starch-less mutant |
|  | CC4334 | TAP, N- | 0h, 6h, 12h, 24h, 48h, 72h, 96h | Starch-less mutant |
|  | CC4565 | TAP, N- | 0h, 6h, 12h, 24h, 48h, 72h, 96h | CC4333 complemented strain |
|  | CC4566 | TAP, N- | 0h, 6h, 12h, 24h, 48h, 72h, 96h | CC4333 complemented strain |
| *Nannochloropsis oceanica* | IMET1 | f/2, N- | 0h, 6h, 12h, 24h, 36h, 48h, 72h, 96h, 120h | Wild-type |
| *Saccharomyces cerevisiae* | Y50049 | YPD | 0h, 3h, 6h, 12h, 24h, 36h, 48h, 72h, 96h, 120h | Model eukaryotic microorganism |
| *Escherichia coli* | DH5a | LB, kan | 0min, 5min, 10min, 20min, 30min, 1h, 3h, 5h | Model prokaryotic microorganism |

**Table S1B**. Assignments of the Raman bands used for tracking the product profile in this study.

| **Raman Peaks** | **Assignments** | **Components** |
| --- | --- | --- |
| 622 | C-C twisting mode of phenylalanine | proteins |
| 643 | C-S stretching & C-C twisting of proteins | proteins |
| 669 | G, T | nucleic acids |
| 725 | characteristic for phospholipids | lipids |
| 758 | Tryptophan, δ (ring) | proteins |
| 783 | U, T, C | nucleic acids |
| 814 | C5’ -O-P-O-C3’ phosphodiester | nucleic acids |
| 865 | C-C-H and C-O-C deformations | carbohydrates |
| 940 | C-O stretching; C-O-C and C-O-H deformation; α-helix C-C backbone | carbohydrates |
| 971 | ν(C-C) wagging | lipids |
| 1003 | Phenylalanine ring breath | proteins |
| 1033 | υ(CO), υ(CC), υ(CCO) | carbohydrates |
| 1045 | C-O and C-C stretching; C-O-H deformation | carbohydrates |
| 1083 | Typical phospholipids | lipids |
| 1127 | C-O stretching | carbohydrates |
| 1176 | C-H in-plane bending mode of tyrosine, (CH) phenylalanine | proteins |
| 1211 | υ(C-C6H5), tryptophan, phenylalanine | proteins |
| 1246 | Amide III | proteins |
| 1265 | Alkyl =C-H | lipids |
| 1305 | CH3/CH2 twisting or bending mode of lipid | lipids |
| 1441 | Alkyl C-H2 bend | lipids |
| 1450 | CH2 bending and scissoring modes of phospholipids | lipids |
| 1481 | Nucleotide acid purine bases | nucleic acids |
| 1575 | Ring breathing modes in the DNA bases | nucleic acids |
| 1584 | Phenylalanine | proteins |
| 1606 | Phenylalanine, tyrosine, C=C | proteins |
| 1619 | υ(C=C), tryptophan | proteins |
| 1658 | Allyl C=C stretches | lipids |
| 1742 | Ester C=O stretches | lipids |
